# New Staphylinidae (Coleoptera) records with new collection data from New Brunswick, and an addition to the fauna of Quebec, Canada: Aleocharinae

**DOI:** 10.3897/zookeys.186.2655

**Published:** 2012-04-26

**Authors:** Reginald P. Webster, Jan Klimaszewski, Jon D. Sweeney, Ian DeMerchant

**Affiliations:** 1 Natural Resources Canada, Canadian Forest Service - Atlantic Forestry Centre, 1350 Regent St., P.O. Box 4000, Fredericton, NB, Canada E3B 5P7; 2 Natural Resources Canada, Canadian Forest Service, Laurentian Forestry Centre, 1055 du P.E.P.S., P.O. Box 10380, Stn. Sainte-Foy, Québec, Quebec, Canada G1V 4C7

**Keywords:** Staphylinidae, Aleocharinae, new records, Canada, New Brunswick

## Abstract

Thirty-eight species of Aleocharinae are newly reported from New Brunswick, bringing the total number of species known from the province to 216. Thirty-one of these species are newly recorded for the Maritime provinces, and four of them, *Phloeopora oregona* Casey, *Gyrophaena michigana* Seevers, *Gyrophaena wisconsinica* Seevers, and *Tomoglossa decora* (Casey), are newly recorded for Canada. *Tomoglossa* constitutes a new generic record for Canada. Collection and habitat data for all these species are presented and discussed. Color habitus, median lobe of the aedeagus, and male tergite and sternite 8 images are presented for the first time for *Phloeopora oregona*, and references to illustrations are provided for all other species included in this paper. A color habitus image is presented for *Tomoglossa decora*.

## Introduction

The Aleocharinae is the largest subfamily of Staphylinidae with over 400 species in 92 genera recorded from Canada (Gouix and Klimaszewski 2007; Brunke et al. 2011). They are morphologically and ecologically diverse, occurring in almost all terrestrial habitats from the intertidal zone of oceans to the alpine zone (Newton et al. 2000). However, species in this subfamily are poorly documented in Canada, and many remain to be discovered and described. In recent years, there has been a dramatic increase in the knowledge of the Aleocharinae fauna of the Maritime provinces (New Brunswick, Nova Scotia, Prince Edward Island). Only 19 species of Aleocharinae were reported from New Brunswick by Campbell and Davies in 1991. Since then, 159 aleocharine species have been added to the provincial list of New Brunswick as a result of new provincial records and new species descriptions, most from publications by Klimaszewski et al. (2003, 2004, 2005, 2006, 2007a, 2008a,b,c, 2009a,b,c, 2011), Assing (2008), Majka and Klimaszewski (2008), Webster et al. (2009), and Majka and Klimaszewski (2010). Majka and Klimaszewski (2010) summarized the history of additions to the aleocharine fauna of the Maritime provinces, added some new provincial records, and presented an updated list of species known from the three Maritime provinces. Currently, 178 species of Aleocharinae have been recorded from New Brunswick. Recent and intensive collecting by the first author and others has resulted in the discovery of many additional species for New Brunswick. In this paper, we report 38 species new to the province, including four new to Canada, bringing the number of species known from the province to 216.

## Methods and conventions

The following records are based in part on specimens collected as part of a general survey by the first author to document the Coleoptera fauna of New Brunswick.

### Collection methods

Various methods were employed to collect the specimens reported in this study. Details are outlined in Campbell (1973) and Webster et al. (2009, Appendix). Some specimens were collected from Lindgren funnel trap samples during a study to develop a general attractant for the detection of invasive species of Cerambycidae. These traps visually mimic tree trunks and are often effective for sampling species of Coleoptera that live in microhabitats associated with standing trees (Lindgren 1983). See Webster et al. (2012) for details of the methods used to deploy Lindgren traps and for sample collection. A description of the habitat was recorded for all specimens collected during this survey. Locality and habitat data are presented exactly as on labels for each record. This information, as well as additional collecting notes, is summarized and discussed in the collection and habitat data section for each species.

### Specimen preparation

Most specimens were dissected to confirm their identity. The genital structures were dehydrated in absolute alcohol and mounted in Canada balsam on celluloid microslides and pinned with the specimens from which they originated.

### Distribution

Distribution maps, created using ArcMap and ArcGIS, are presented for each species in New Brunswick. Every species is cited with current Distribution in Canada and Alaska, using abbreviations for the state, provinces, and territories. New provincial records are indicated in bold under Distribution in Canada and Alaska. The following abbreviations are used in the text:

**Table:** 

**AK**	Alaska	**MB**	Manitoba
**YT**	Yukon Territory	**ON**	Ontario
**NT**	Northwest Territories	**QC**	Quebec
**NU**	Nunavut	**NB**	New Brunswick
**BC**	British Columbia	**PE**	Prince Edward Island
**AB**	Alberta	**NS**	Nova Scotia
**SK**	Saskatchewan	**NF & LB**	Newfoundland and Labrador*

* Newfoundland and Labrador are each treated separately under the current Distribution in Canada and Alaska.

Acronyms of collections examined and referred to in this study are as follows:

**AFC** Atlantic Forestry Centre, Natural Resources Canada, Canadian Forest Service, Fredericton, New Brunswick, Canada

**CNC** Canadian National Collection of Insects, Arachnids and Nematodes, Agriculture and Agri-Food Canada, Ottawa, Ontario, Canada

**LFC **Laurentian Forestry Centre, Natural Resources Canada, Canadian Forest Service, Ste. Foy, Quebec, Canada

**NBM** New Brunswick Museum, Saint John, New Brunswick, Canada

**RWC** Reginald P. Webster Collection, Charters Settlement, New Brunswick, Canada

## Results

### Species accounts

All records below are species newly recorded for New Brunswick, Canada. Species with ** are newly recorded from the Maritime provinces; species with *** are newly recorded for Canada.

#### Family Staphylinidae Latreille, 1806

**Subfamily Aleocharinae Fleming, 1821**

**Tribe Aleocharini Fleming, 1821**

##### Aleochara rubripennis

(Casey, 1906)**

http://species-id.net/wiki/Aleochara_rubripennis

**Map 1.**
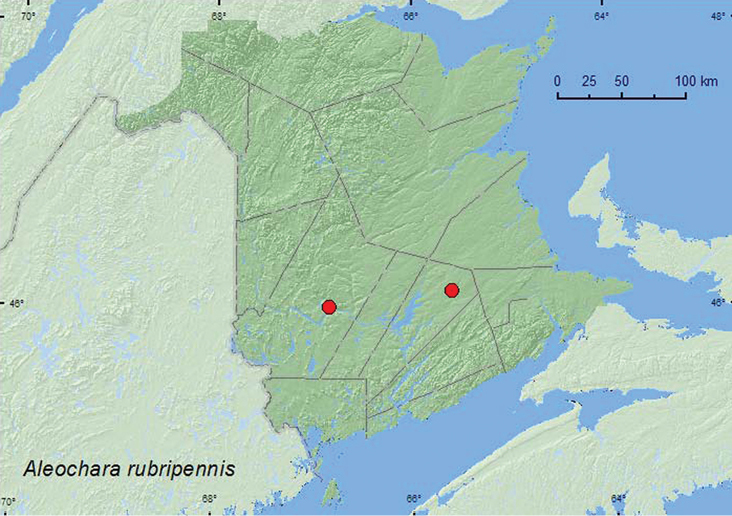
Collection localities in New Brunswick, Canada of *Aleochara rubripennis*.

Map 1 illustrations in Klimaszewski (1984)

###### Material examined.

**New Brunswick, Queens Co.**, Cranberry Lake P.N.A. (Protected Natural Area), 46.1125°N, 65.6075°W, 21–27. V.2009, R. Webster & M.-A. Giguère, mature red oak forest, Lindgren funnel trap (1 ♀, RWC). **York Co.**, Keswick River at Rt. 105, 45.9938°N, 66.8344°W, 3.VI.2008, R. P. Webster, silver maple swamp, in entrance to woodchuck burrow (1 ♀, RWC).

###### Collection and habitat data.

This species was reported from groundhog (*Marmota* sp.) and ground squirrel (*Cittellus* sp.) burrows, usually early in the spring (Klimaszewski 1984). Adults were taken from moist soil and grass roots in or near the entrances to the burrows. One of the specimens from New Brunswick was collected from litter in the entrance to a groundhog or woodchuck (*Marmota monax* (L.)) burrow. The other individual was captured in a Lindgren funnel trap deployed in an old red oak (*Quercus rubra* L.) forest. Adults were collected during May and early June.

###### Distribution in Canada and Alaska.

MB, ON, QC, **NB** (Klimaszewski 1984; Gouix and Klimaszewski 2007).

#### Tribe Oxypodini Thomson, 1859

##### Gnathusa minutissima

Klimaszewski & Langor 2011**

http://species-id.net/wiki/Gnathusa_minutissima

**Map 2.**
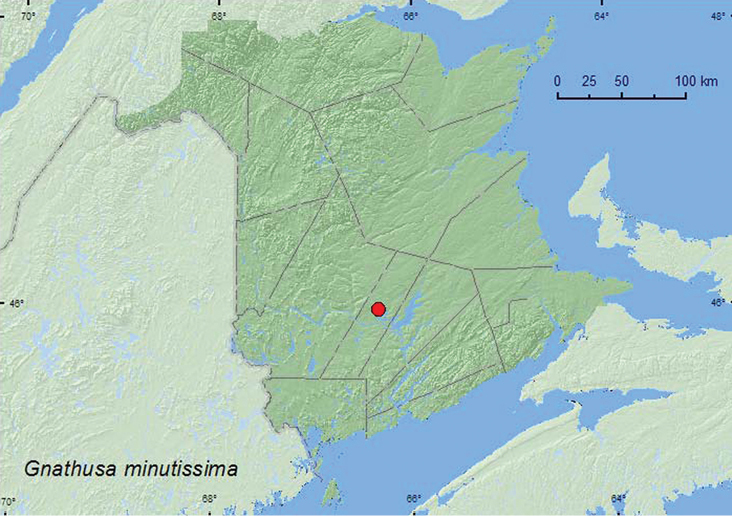
Collection localities in New Brunswick, Canada of *Gnathusa minutissima*.

Map 2 illustrations in Klimaszewski et al. (2011)

###### Material examined.

**New Brunswick, Sunbury Co.,** Acadia Research Forest, 45.9799°N, 66.3394°W, 18.VI.2007, R. P. Webster coll., mature red spruce and red maple forest, sifting leaf litter (1 ♀, 1 ♂, RWC, LFC); same locality data and collector except 14.V.2007, sifting moss near brook (1 ♀, 1 ♂, RWC, LFC).

###### Collection and habitat data.

In New Brunswick, adults were found in leaf litter and moss near a brook in a mature red spruce (*Picea rubens* Sarg.) and red maple (*Acer rubrum* L.) forest. In Newfoundland, adults were collected in pitfall traps in an old balsam fir (*Abies balsamea* (L.) Mill.) forest in June and July (Klimaszewski et al. 2011).

###### Distribution in Canada and Alaska

**.** NF, **NB** (Klimaszewski et al. 2011).

##### Oxypoda orbicollis

Casey, 1911

http://species-id.net/wiki/Oxypoda_orbicollis

**Map 3.**
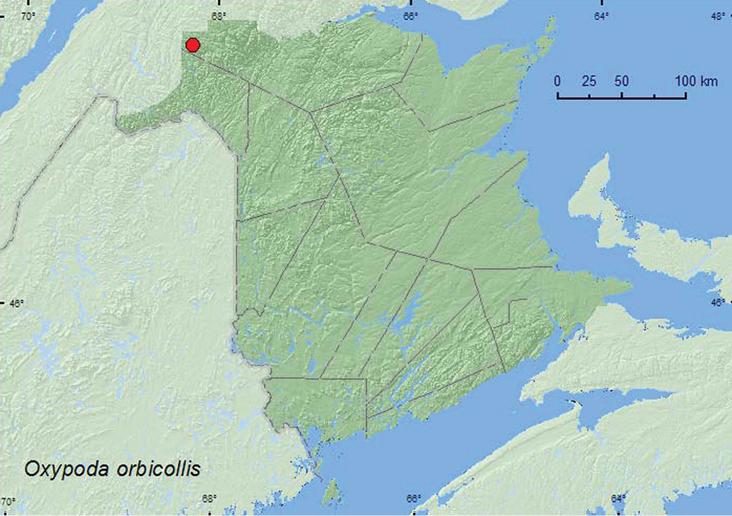
Collection localities in New Brunswick, Canada of *Oxypoda orbicollis*.

Map 3 illustrations in Klimaszewski et al. (2006)

###### Material examined.

**New Brunswick, Restigouche Co.**, Mount Atkinson, 447 m elev., 47.8192°N, 68.2618°W, 21.VII.2010, R. P. Webster, boreal forest, small shaded spring-fed brook with mossy margin, sifting saturated moss (1 ♀, RWC).

###### Collection and habitat data.

In eastern Canada, adults were found in balsam fir forests and maple forests and were collected in pitfall traps, Luminoc pitfall traps, and Lindgren funnel traps or sifted from forest litter and sphagnum (Klimaszewski et al. 2006). The specimen from New Brunswick was sifted from saturated moss on the margin of a spring-fed brook in a balsam fir and white spruce (*Picea glauca* (Moench) Voss) forest during July.

###### Distribution in Canada and Alaska.

YT, AB, ON, QC, **NB**, NS, LB (Klimaszewski et al. 2006; Klimaszewski et al. 2011).

##### Phloeopora oregona

Casey, 1906***

http://species-id.net/wiki/Phloeopora_oregona

Map 4 Figs 1–4

###### Material examined.

**Canada, New Brunswick, York Co.,** Charters Settlement, 45.8340°N, 66.7450°W, 14.V.2004, R. P. Webster coll., mixed forest, in wood pile under bark of spruce (1 ♂, 1 sex undetermined, RWC); same data except 45.8188°N, 66.7460°W, 16.IV.2005, R. P. Webster coll., clearcut, under bark of white pine log (1 ♂, LFC); same locality data and collector except 45.8286°N, 66.7365°W, 3.VI.2007, 6.VI.2007, mature red spruce and red maple forest, under scolytid infested bark of red spruce (1 ♂, 2 ♀, 1 sex undetermined, RWC); 15 km W of Tracy, off Rt. 645, 45.6848°N, 66.8821°W, 26.IV–0.V.2010, R. Webster & C. MacKay, coll., old red pine forest, Lindgren funnel trap (1 ♀, RWC).

**Map 4.**
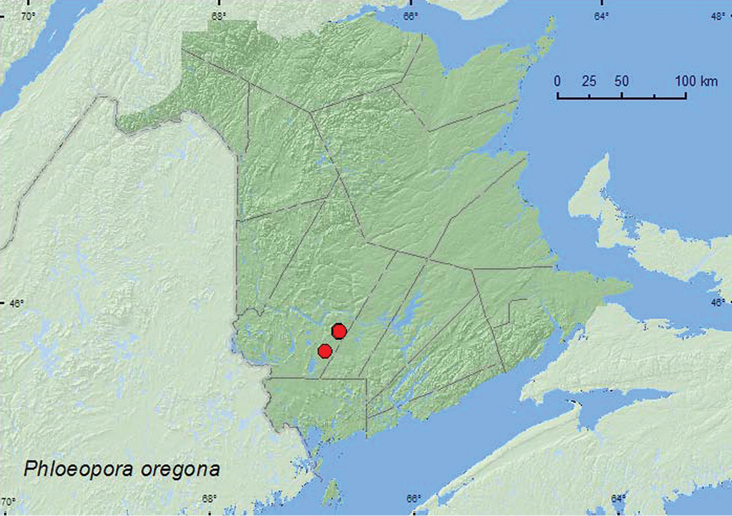
Collection localities in New Brunswick, Canada of *Phloeopora oregona*.

**Figures 1–4.**
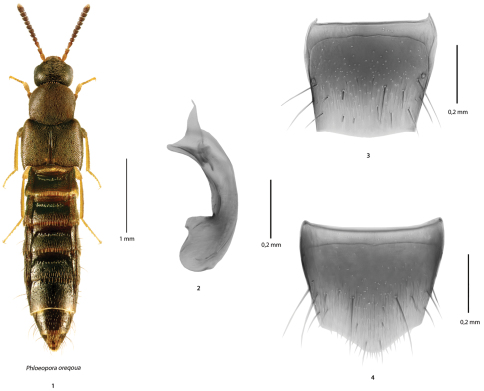
*Phloeopora oregona* Casey (based on male specimens from NB): **1** habitus in dorsal view **2** median lobe of the aedeagus in lateral view **3** tergite 8 and **4** sternite 8.

###### Collection and habitat data.

In New Brunswick, adults were found in a mixed forest, a mature red spruce (*Picea rubens* Sarg.) and red maple forest, and in an old red pine (*Pinus resinosa* Ait.) forest. Specimens were taken from under bark of spruce and white pine (*Pinus strobus* L.), and under bark of red spruce infested with Scolytinae. One individual was captured in a Lindgren funnel trap. Specimens were collected during April, May, and June.

###### Distribution in Canada and Alaska.

**NB** (new Canadian record).This specieswas, until now, only known from the type locality (The Dalles) in Oregon (Casey 1906). We suspect that it is broadly distributed and transcontinental in North America. It is rare in collections, probably due to cryptic habitat associations.

###### Comments.

Author JK examinedthetype material ofthe following species: *Phloeopora arctica* Lohse, *Phloeopora corticalis* (Gravenhorst), *Phloeopora ferruginea* Casey, *Phloeopora liberta* Casey, *Phloeopora oregona* Casey, *Phloeopora sublaevis* Casey, *Phloeopora scriba* Eppelsheim, and *Phloeopora testacea* (Mannerheim). All species of this genus are similar externally to each other and differ in small details such as body proportions (e.g., width of pronotum, length of elytra), density of punctation and pubescence on forebody, body color, and shape of the apical part of median lobe of aedeagus in lateral view. The shape of the median lobe of aedeagus in specimens from New Brunswick is similar to that of Palaearctic *Phloeopora corticalis* and Nearctic *Phloeopora oregona*, but externally is more similar to *Phloeopora oregona* and *Phloeopora testacea*, which have dense pronotal punctation and pubescence, and are less glossy than *Phloeopora corticalis*. The median lobe of aedeagus is strongly produced ventrally at apex in *Phloeopora corticalis* and *Phloeopora oregona* and less so in the other species. We tentatively affiliate the New Brunswick specimens with *Phloeopora oregona* and suspect that this species is transcontinental in distribution in North America.

##### Brachyusa helenae

(Casey 1911)**

http://species-id.net/wiki/Brachyusa_helenae

**Map 5.**
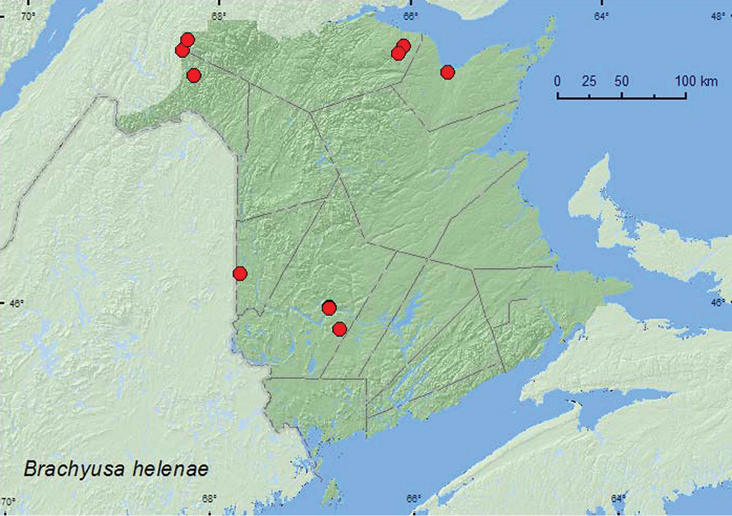
Collection localities in New Brunswick, Canada of *Brachyusa helenae*.

Map 5 illustrations in Klimaszewski et al. (2011)

###### Material examined.

**New Brunswick, Carleton Co.,** Jackson’s Falls, 46.2257°N, 67.7437°W, 12.IX.2009, R. P. Webster coll., river margin near waterfall, splashing moss near splash zone of waterfall (10 ♂, 8 ♀, RWC, LFC); **Gloucester Co.**, Bathurst, Daly Point Reserve, 27.VII.2009, R. P. Webster, sea beach, in seepage area (fresh water) (1 ♀, LFC). **Madawaska Co.**, Third Lake, 47.7786°N, 68.3783°W, 21.VI.2010, R. P. Webster, partially shaded brook, gravel/clay margin, under alders (splashing & turning gravel) (1 ♂, LFC); Gagné Brook at First Lake Rd., 47.6077°N, 68.2534°W, 23.VI.2010, M. Turgeon & R. P. Webster, northern hardwood forest, shaded brook, among gravel on gravel bars, splashing & turning gravel (1 ♂, LFC). **Restigouche Co.**, Jacquet River Gorge P.N.A., 47.8256°N, 66.0770°W, 13.VIII.2010, R. P. Webster, large shaded brook among cobblestones (1, sex undetermined, NBM); same locality and collector except 47.7765°N, 66.1277°W, 13.VIII.2010, Jacquet River, among moss on rocks in middle of river, collected by splashing rocks (1 ♀, RWC); Wild Goose Lake, 420 m elev., 47.8540°N, 68.3219°W, 7.VI.2011, R. P. Webster & M. Turgeon, lake margin with emergent *Carex* & grasses, treading *Carex* & grasses (1 sex undetermined, LFC). **York Co.**, Keswick River at Rt. 105, 45.9938°N, 66.8344°W, 3.VI.2008, R.P. Webster coll., upper river margin, in flood debris on sand/clay mix (2 ♀, RWC); Keswick River at Rt. 105, 45.9920°N, 66.8334°W, 9.VII.2009, silver maple swamp, margin of vernal pond, splashing (1 ♀, NBM); Charters Settlement, 45.8391°N, 66.7345°W, 25.IV.2010, R. P. Webster, beaver dam, in debris near outflow from dam (1 sex undetermined, LFC).

###### Collection and habitat data.

In New Brunswick, most adults of *Brachyusa helenae* were found near flowing water. Many specimens were collected frommoss near the splash zone of a waterfall. It took 5–10 min. before adults appeared after repeated splashing. Other individuals were collected from moss on rocks in the middle of a river, from gravel on a gravel bar along a shaded brook, from gravel on a gravel/clay margin of a partially shaded brook, from among cobblestones along a large shaded brook, and in flood debris resting on a sand/clay mix along an upper river margin. Most individuals from these habitats were collected by splashing water on moss and gravel, or turning gravel. A few specimens were collected by treading emergent *Carex* and grasses on the margin of a lake near the outflow of a stream and by splashing water on debris on the margin of a vernal pond in a silver maple (*Acer saccharinum* L.) swamp near a river. One individual was sifted from debris on a beaver (*Castor canadensis* Kuhl) dam near the outflow area (flowing water) from the dam. Adults from New Brunswick were collected during April, June, July, August, and September. In Labrador, adults were captured in July and August on sand and gravel on the banks of the Churchill River (Klimaszewski et al. 2011). Elsewhere, adults were collected near lake and river shorelines, on clay, sand and gravel beaches, and silty river margins (Klimaszewski et al. 2011).

###### Distribution in Canada and Alaska.

AK**,** NT, **NB**, LB, NF (Klimaszewski et al. 2011).

##### Gnypeta atrolucens

Casey, 1894**

http://species-id.net/wiki/Gnypeta_atrolucens

**Map 6.**
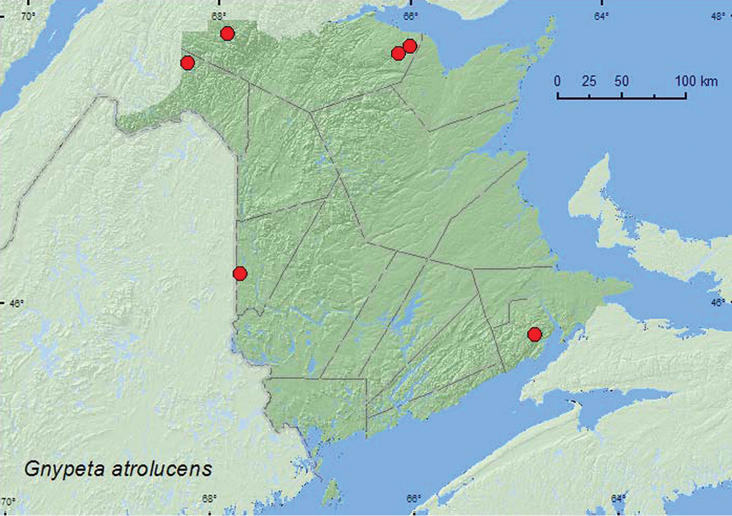
Collection localities in New Brunswick, Canada of *Gnypeta atrolucens*.

Map 6 illustrations in Klimaszewski et al. (2008c)

###### Material examined.

**New Brunswick, Albert Co.,** Caledonia Gorge P.N.A., at Crooked Creek, 45.7930°N, 64.7764°W, 1.VII.2011, R. P. Webster, small clear cold rocky river, in moss on rocks on river margin (1 ♂, 1 ♀, NBM). **Carleton Co.** Jackson Falls, 46.2257°N, 67.7437°W, 12.IX.2009, R. P. Webster, river margin near waterfall, splashing moss near splash zone of waterfall (2 ♂, 1 ♀, RWC). **Madawaska Co.**, at Green River, 47.6918°N, 68.3202°W, 21.VI.2010, M. Turgeon & R. Webster, river margin, among gravel on gravel bar (1 ♀, RWC). **Restigouche Co.**, Kedgwick Forks, 47.9085°N, 67.9057°W, 22.VI.2010, R. P. Webster, on exposed rocks in middle of river (1 ♂, NBM); Jacquet River Gorge P.N.A., 47.7765°N, 66.1277°W, 13.VIII.2010, R. P. Webster, Jacquet River, among moss on rocks in middle of river, collected by splashing rocks (2 ♂, 2 ♀, NBM, RWC); same locality and collector but 47.8208°N, 66.0088°W, 14.VIII.2010, shaded brook, in moss on rock in middle of brook (1 ♂, NBM).

###### Collection and habitat data.

In New Brunswick, *Gnypeta atrolucens* was typically found in riparian habitats. Adults were collected by splashing water on moss near the splash zone of a waterfall, splashing water on moss and rocks in the middle of a river and a brook, and splashing water on exposed rocks in the middle of a river. At the latter site, adults emerged from cracks in the rocks after splashing. It generally took up to 10 min. and repeated splashing before adults appeared on the moss or rocks. One individual was collected from gravel on a gravel bar on a river margin. Elsewhere, adults were collected during July and August at altitudes from 61–853 m, otherwise little was previously known about the habitat associations of this species (Klimaszewski et al. 2008c).

###### Distribution in Canada and Alaska.

QC, **NB**, NF, LB (Klimaszewski et al. 2008c, 2011).

##### Tachyusa americanoides

Paśnik, 2006

http://species-id.net/wiki/Tachyusa_americanoides

**Map 7.**
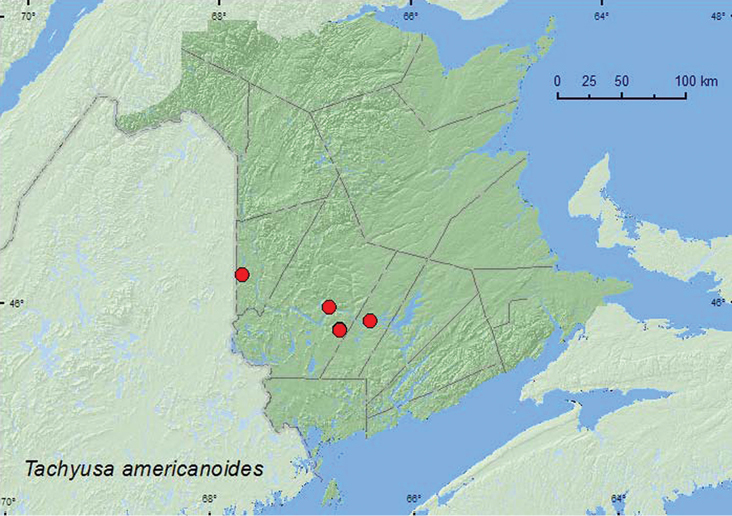
Collection localities in New Brunswick, Canada of *Tachyusa americanoides*.

Map 7 illustrations in Paśnik (2006) Klimaszewski et al. (2011)

###### Material examined.

**New Brunswick, Carleton Co.,** Wakefield, Bell Forest Nature Preserve, 46.2152°N, 67.7190°W, 12.VII.2004, K. Bredin, J. Edsall & R. Webster, coll., river margin, under debris (1 sex undetermined, RWC). **Sunbury Co.**, Maugerville, Portebello Creek N.W.A. (National Wildlife Area), 45.8992°N, 66.4248°W, 24.VI.2004, R.,P. Webster coll., silver maple forest, margin of slow river under litter on muddy soil (1 sex undetermined, 1 ♀, LFC, RWC). **York Co.,** Douglas, Keswick River at Rt. 105, 45.9943°N, 66.8337°W, 18.VI.2004, R. P. Webster, coll., silver maple forest, under debris on muddy soil near small pool (2 sex undetermined, LFC, RWC); Charters Settlement, 45.8456°N, 66.7267°W, 16.V.2010, 10.VI.2010, R. P. Webster, coll., beaver dam, among grassy debris near an overflow area of dam, near flowing water (2 ♂, 1 ♀, RWC); same locality and collector but 45.8331°N, 66.7279°W, 20.V.2010, beaver dam, among sticks, debris and clay on dam (1 ♂, RWC).

###### Collection and habitat data.

In New Brunswick, *Tachyusa americanoides* was collected from grassy debris near the overflow area of a beaver dam, from among sticks, debris, and clay on a beaver dam, from debris on muddy soil along a slow-flowing river in a silver maple swamp, from debris along the margin of a rocky river, and from debris on muddy soil near a small pool in a silver maple forest. Adults were collected during May, June, and July.

###### Distribution in Canada and Alaska.

NT, BC, AB, MB, ON, **NB,** NS, NL (Paśnik 2006; Gouix and Klimaszewski 2007; Klimaszewski et al. 2011). *Tachyusa americanoides* was recorded by Klimaszewski et al. (2011) from NB without specifying locality data. We record this species here from NB for the first time with locality data and habitat information.

##### Tachyusa obsoleta

Casey, 1906**

http://species-id.net/wiki/Tachyusa_obsoleta

**Map 8.**
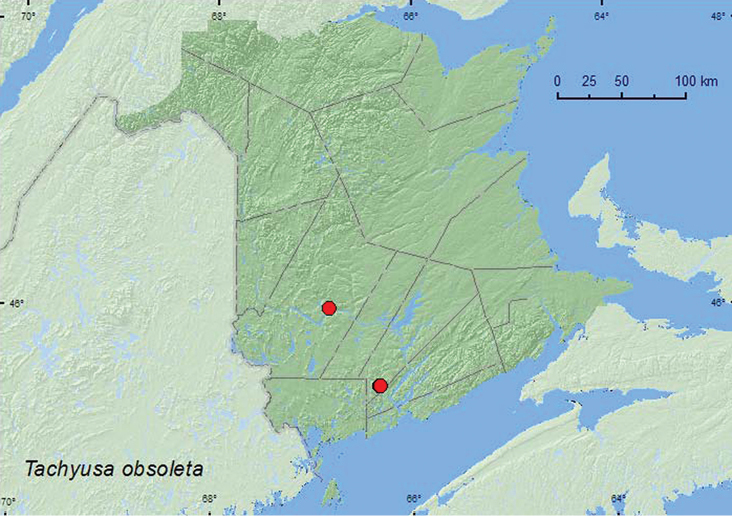
Collection localities in New Brunswick, Canada of *Tachyusa obsoleta*.

Map 8 illustrations in Paśnik (2006)

###### Material examined. 

**New Brunswick, Queens Co.,** Welsford (Bayard) near Nerepis River, 45.4441°N, 66.3300°W, 27.VI.2006, R. P. Webster, coll., river margin, among grass and debris near water (3 ♀, RWC); Bayard at Nerepis river, 45.4426°N, 66.3280°W, 25.V.2008, 30.V.2008, 20.VI.2008, R. P. Webster, coll., river margin, lightly splashing fine sand (3 ♂, 3 ♀, LFC, RWC). **York Co.,** Douglas, Keswick River at Rt. 105, 45.9922°N, 66.8326°W, 9.V.2006, R. P. Webster, river margin, on moist clay (1 ♀, RWC).

###### Collection and habitat data.

In New Brunswick, most adults of *Tachyusa obsoleta* were collected during May and June by lightly splashing water on fine sand near a river margin. Other individuals were found on moist clay and by sifting grass and debris along river margins.

###### Distribution in Canada and Alaska.

BC, SK, **NB** (Paśnik 2006). For records from the USA, see Paśnik (2006). This is almost certainly a transcontinental species in North America.

#### Tribe Homalotini Heer, 1839

##### Gyrophaena nana

(Paykull, 1800)**

http://species-id.net/wiki/Gyrophaena_nana

**Map 9.**
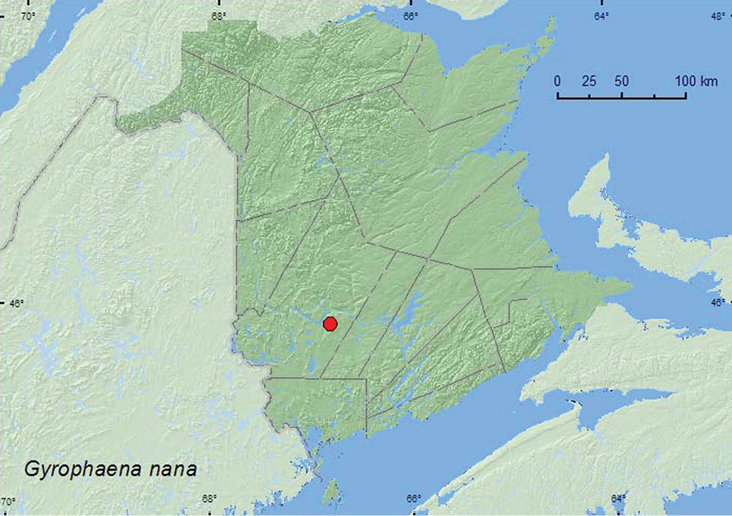
Collection localities in New Brunswick, Canada of *Gyrophaena nana*.

Map 9 illustrations in Klimaszewski et al. (2011)

###### Material examined.

**New Brunswick, York Co.,** NE of Exit 271 off Hwy 2, 45.8776°N, 66.8254°W, 8.VI.2008, Stephen Clayden, coll., mixed forest, in mushroom on log (1 ♂, RWC).

###### Collection and habitat data.

The specimen from New Brunswick was collected from a mushroom in a mixed forest during June.

###### Distribution in Canada and Alaska.

YT, AK, BC, AB, MB, ON, **NB,** NF (Gouix and Klimaszewski 2007; Klimaszewski et al. 2009b, 2011).

##### Gyrophaena neonana

Seevers, 1951**

http://species-id.net/wiki/Gyrophaena_neonana

**Map 10.**
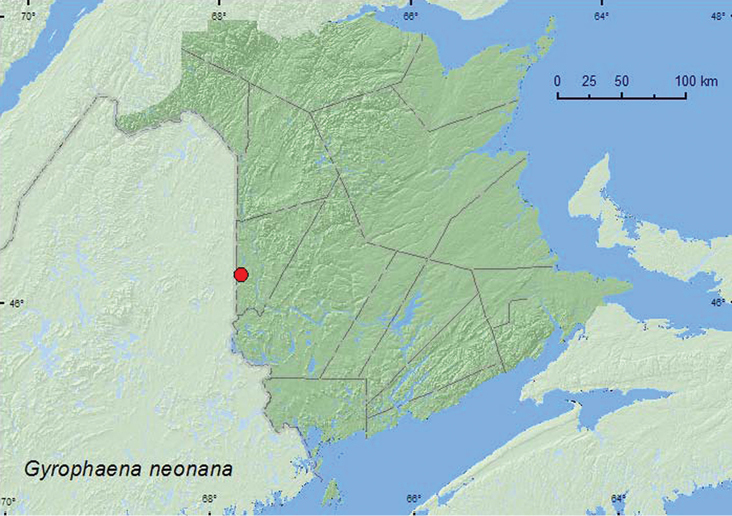
Collection localities in New Brunswick, Canada of *Gyrophaena neonana*.

Map 10 illustrations in Klimaszewski et al. (2011)

###### Material examined.

**New Brunswick, Carleton Co.**, Jackson Falls, 46.2200°N, 67.7230°W, 12.IX.2008, R. P. Webster, hardwood forest, in fleshy polypore mushroom on beech log (1 ♂, RWC).

###### Collection and habitat data.

The sole specimen from New Brunswick was collected from a fleshy polypore mushroom on an American beech (*Fagus grandifolia* Ehrh.) log during September. Little is known about the bionomics of this species.

###### Distribution in Canada and Alaska.

YT, **NB**, NF (Gouix and Klimaszewski 2007; Klimaszewski et al. 2011).

##### Gyrophaena caseyi

Seevers, 1951**

http://species-id.net/wiki/Gyrophaena_caseyi

**Map 11.**
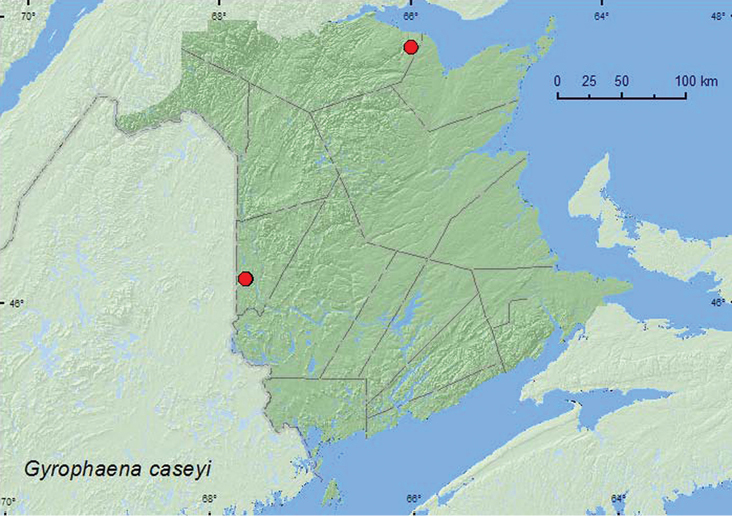
Collection localities in New Brunswick, Canada of *Gyrophaena caseyi*.

Map 11 illustrations Seevers (1951)

###### Material examined.

**New Brunswick, Carleton Co.**, Meduxnekeag Valley Nature Preserve, 46.1910°N, 67.6740°W, 13.VIII.2006, R. P. Webster, mixed forest, on *Pleurotus* sp. on side of log (1 ♂, RWC); near Belleville, 1.3 km E jct. Rt. 540 & Plymouth Rd., 46.1880°N, 67.6848°W, 20.IX.2008, R. P. Webster, hardwood forest, in small gilled mushrooms on log (2 ♂, 4 ♀, RWC, 2 ♂, LFC). **Restigouche Co.**, Jacquet River Gorge P. N. A., 47.8201°N, 65.9992°W, 12.VIII.2010, R. P. Webster, black spruce/balsam fir/cedar forest near Belledune Bog, in gilled mushroom (1 ♂, RWC).

###### Collection and habitat data.

In New Brunswick, adults of *Gyrophaena caseyi* were collected during August and September from fresh gilled mushrooms and *Pleurotus* sp. on the side of a log. This species was found in a hardwood forest, a mixed forest, and a black spruce (*Picea mariana* (Mill.) B.S.P.), balsam fir, and eastern white cedar (*Thuja occidentalis* L.) forest.

###### Distribution in Canada and Alaska

**.** QC, **NB** (Klimaszewski et al. 2009b).

###### Comment.

*Gyrophaena caseyi* and *Gyrophaena nanoides* Seevers are very similar to each other externally and have similar genitalia. These two species were confused by Klimaszewski et al. (2009b), and specimens recorded from New Brunswick as *Gyrophaena caseyi* were *Gyrophaena nanoides*. This error and differences between these two species were pointed out by Klimaszewski et al. (2011). Specimens of *Gyrophaena caseyi* were found in New Brunswick since the publication of Klimaszewski et al. (2009b), and these represent a new provincial record. Collection data, habitat notes, and distributional maps are presented for both species.

##### Gyrophaena nanoides

Seevers, 1951**

http://species-id.net/wiki/Gyrophaena_nanoides

**Map 12.**
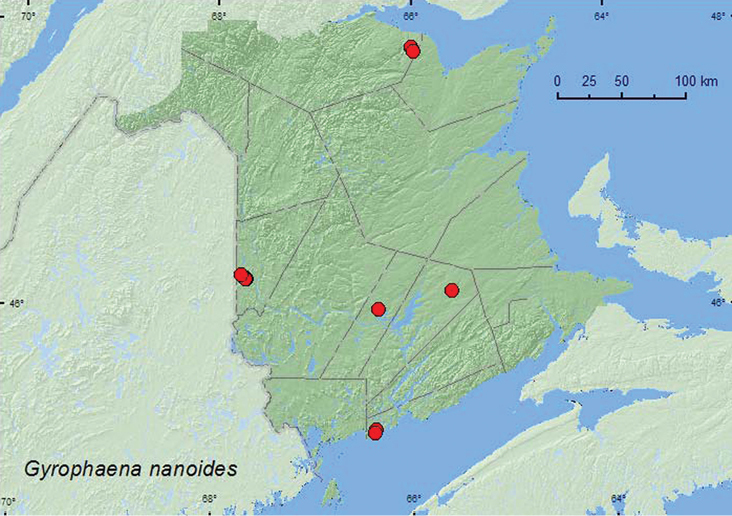
Collection localities in New Brunswick, Canada of *Gyrophaena nanoides*.

Map 12 illustrations Klimaszewski et al. (2011)

###### Material examined.

**New Brunswick, Carleton Co.**, (Belleville) Meduxnekeag Valley Nature Preserve, 46.1980°N, 67.6854°W, 31.VIII.2006, R. P. Webster, mixed forest, on gilled mushroom(1 ♂, 2 ♀, NBM, RWC); same locality and collector but 46.1907°N, 67.6740°W, 7.IX.2004, mixed forest, on fleshy (gilled) fungi (1 ♂, 1 ♀, NBM); same locality and collector but 46.1897°N, 67.6710°W, 12.IX.2008, mixed forest, on gilled mushroom, (GYR-RW-8, 1 ♂, LFC); near Belleville, 1.3 km E jct. Rt. 540 & Plymouth Rd., 46.1860°N, 67.6847°W, 20.IX.2008, R. P. Webster, mixed forest with hemlock, on small gilled mushrooms on rotten log (GYR-RW-22, 1 ♂, LFC; GYR-RW-23, 1 ♂, NBM, GYR-RW-24, 1 ♂, NBM; GYR-RW-25, 1 ♂, NBM ); same locality data and collector but 20.IX.2008, on *Pleurotus* sp. on log (GYR-RW-61, 1 ♂, NBM); Jackson Falls, “Bell Forest”, 46.2200°N, 67.7230°W, 12.IX.2008, R. P. Webster, hardwood forest, on gilled mushroom on log (GYR-RW-36, 1 ♂, NBM; GYR-RW-37, 1 ♂, NBM; GYR-RW-35, 1 ♀, NBM; GYR-RW-27, 1 sex undetermined, NBM). **Queens Co.**, Cranberry Lake P.N.A., 46.1125°N, 65.6075°W, 2.IX.2009, R. P. Webster, red oak forest, in small stalked polypore fungus on forest floor (1 ♂, RWC); same locality data, forest type, and collector, 22.IX.2009, in *Boletus* sp. (2 ♂, RWC). **Restigouche Co.**, Jacquet River Gorge P.N.A., 47.8201°N, 65.9992°W, 12.VIII.2010, R. P. Webster, black spruce/balsam fir/cedar forest near Belledune Bog, in gilled mushroom (1 ♂, RWC); same locality and collector but 47.7883°N, 65.9819°W, 17.VIII.2010, black spruce forest, mossy forest floor, in *Russula* mushroom (2 ♂, NBM, RWC). **Saint John Co.**, Chance Harbour, 45.1391°N, 66.3696°W, 24.VIII.2006, R. P. Webster, red spruce and birch forest, on gilled mushrooms (1 ♂, 1 ♀, LFC; 1 ♂, NBM; 2 ♂, RWC; Photo 2008-84, 1 ♂, LFC; Photo 2008-85, 1 ♀, LFC); same locality data and collector but 16.IX.2008, yellow birch & spruce forest, on gilled mushrooms on forest floor (GYR-RW-71, 1 ♂, NBM; GYR-RW-77, 1 ♂, NBM; GYR-RW-74, 1 ♂, LFC); Dipper Harbour, 45.1176°N, 66.3806°W, 12.IX.2006, R. P. Webster, red spruce forest, on gilled mushrooms (1 ♂, LFC; 2 ♂, RWC). **Sunbury Co.** Acadia Research Forest, 45.9799°N, 66.3394°W, 18.IX.2007, R. P. Webster, Road 7 control, mature red spruce and red maple forest, in gilled mushroom (1 ♂, AFC).

###### Collection and habitat data.

In New Brunswick, *Gyrophaena nanoides* was found in various deciduous and coniferous forest types, including hardwood forests with sugar maple (*Acer saccharum* Marsh.), American beech, and white ash (*Fraxinus americana* L.), an old red oak forest, a mixed forest with eastern hemlock (*Tsuga canadensis* (L.) Carr.), mixed forests, red spruce forests, a black spruce forest, and a black spruce, balsam fir and eastern white cedar forest. Most adults were collected from fresh (not decaying) gilled mushrooms, including a *Russula* sp. Some specimens were collected from a stalked polypore fungus on forest floor, a *Boletus* sp., and a *Pleurotus* sp. on a log. Little was previously known about the habitat associations and bionomics of this species. Adults were collected during August and September.

###### Distribution in Canada and Alaska.

ON,QC, **NB**, NF (Klimaszewski et al. 2009b, 2011).

#### *Gyropheana gaudens* species group, *sensu* Seevers, 1951

*Gyrophaena gaudens* Casey, *Gyrophaena michigana* Seevers, and *Gyrophaena uteana* Caseyare very similar to each other externally and have similar genitalia. These species were confused by Klimaszewski et al. (2009b), and specimens recorded from New Brunswick as *Gyrophaena gaudens*are *Gyrophaena uteana*. *Gyrohphaena gaudens* is accordingly removed from the faunal list of New Brunswick. Specimens recorded from New Brunswick as *Gyrophaena uteana* are *Gyrophaena michigana* Seevers, a species new to the province and Canada. Accordingly, new distributional maps and collection and habitat data are presented below for *Gyrophaena michigana* and *Gyrophaena uteana*.

##### Gyrophaena michigana

Seevers, 1951***

http://species-id.net/wiki/Gyrophaena_michigana

**Map 13.**
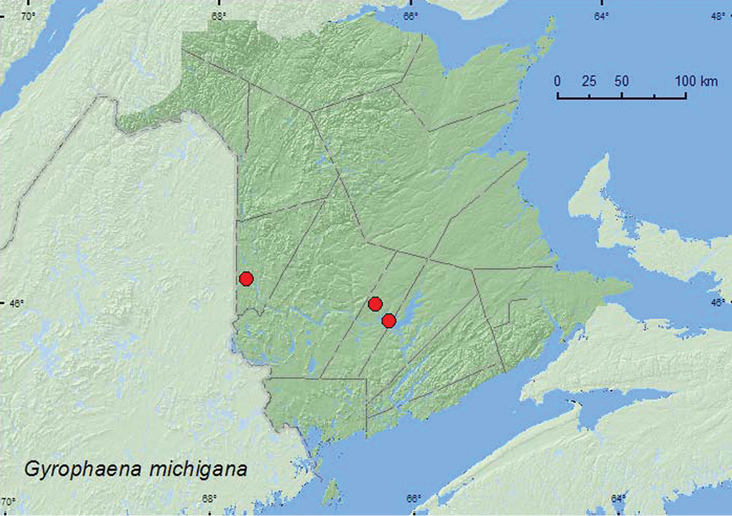
Collection localities in New Brunswick, Canada of *Gyrophaena michigana*.

Map 13 Figs 116e,f in Seevers (1951) Figs 145–151 in Klimaszewski et al. (2009b) (erroneously as *Gyrophaena uteana* Casey).

###### Material examined.

**Canada, New Brunswick, Carleton Co.**, Belleville, Meduxnekeag Valley Nature Preserve, 46.1907°N, 67.6740°W, 23.VI.2006, R. P. Webster, mixed forest, on gilled mushrooms (2 ♂, RWC). **Sunbury Co.**, Lakeville Corner, 45.9007°N, 66.2423°W, 12.VII.2006, R. P. Webster, silver maple swamp, on ridge with oaks & red maple, on gilled mushroom (1 ♂, RWC); Acadia Research Forest, 46.0173°N, 66.3741°W, 17.VIII.2007, R. P. Webster, Road 7 Control, mature red spruce & red maple forest, in gilled mushrooms (2 ♂, LFC; 1 ♂, 1 ♀, RWC).

###### Collection and habitat data.

In New Brunswick, this species was collected from fresh gilled mushrooms in a mixed forest, a silver maple swamp, and a mature red spruce and red maple forest. Adults were collected during June, July, and August. Little was previously known about the habitat associations and bionomics of this species.

###### Distribution in Canada and Alaska.

**NB** (new Canadian record). Seevers (1951) reported this species from Michigan, Illinois, and Wisconsin in the United States.

##### Gyrophaena uteana

Casey, 1906**

http://species-id.net/wiki/Gyrophaena_uteana

**Map 14.**
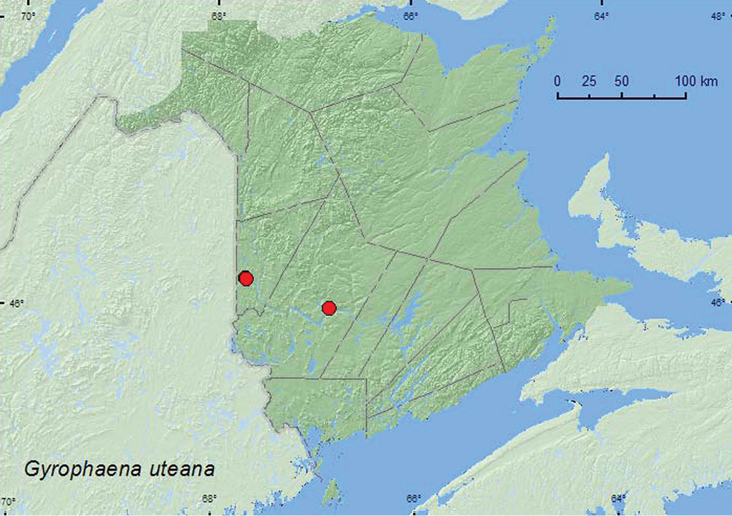
Collection localities in New Brunswick, Canada of *Gyrophaena uteana*.

Map 14 Figs 116c,d in Seevers (1951) Figs 152–158 in Klimaszewski et al. (2009b) (erroneously as *Gyrophaena gaudens* Casey).

###### Material examined.

**New Brunswick , Carleton Co.**, (Belleville) Meduxnekeag Valley Nature Preserve, 46.1957°N, 67.6803°W, 1.VIII.2004, R. P. Webster, mixed forest, on bracket fungi (1 ♂, LFC 1 ♀, RWC); same locality and collector but 46.1907°N, 67.6740°W, 23.VI.2006, mixed forest, on gilled mushroom (1 ♂, RWC); same locality data and collector but 19.VII.2006, mixed forest, on small gilled mushrooms on log (Photo 2008-93, ♂, RWC); same locality data and collector but 7.IX.2004, mixed forest, on fleshy (gilled) fungi (Photo 2008-107, 1 ♀, LFC); same locality and collector but 46.1940°N, 67.6800°W, 3.VII.2006, mixed forest, in *Pleurotus* sp. on dead standing *Populus tremuloides* (Photo 2008-106, 1 ♂, LFC; 1 ♂, RWC); same locality and collector but 46.1910°N, 67.6740°W, 31.VIII.2006, mixed forest, on polypore fungi (1 ♂, LFC; 1 ♂, RWC). **York Co.**, Keswick River at Rt. 105, 45.9920°N, 66.8334°W, 9.VII.2009, R. P. Webster, silver maple swamp, on small gilled mushrooms on log (2 ♂, RWC).

###### Collection and habitat data.

*Gyrophaena uteana* from New Brunswick was collected in mixed forests and a silver maple swamp. Adults were collected from polypore fungi, on fresh gilled mushrooms, on a small (fresh) gilled mushroom on a log, and from a *Pleurotus* sp. on a dead, standing trembling aspen (*Populus tremuloides* Michx.). Adults were collected during June, July, August, and September.

###### Distribution in Canada and Alaska.

BC, QC, **NB** (Klimaszewski et al. 2009b).

##### Gyrophaena wisconsinica

Seevers, 1951***

http://species-id.net/wiki/Gyrophaena_wisconsinica

**Map 15.**
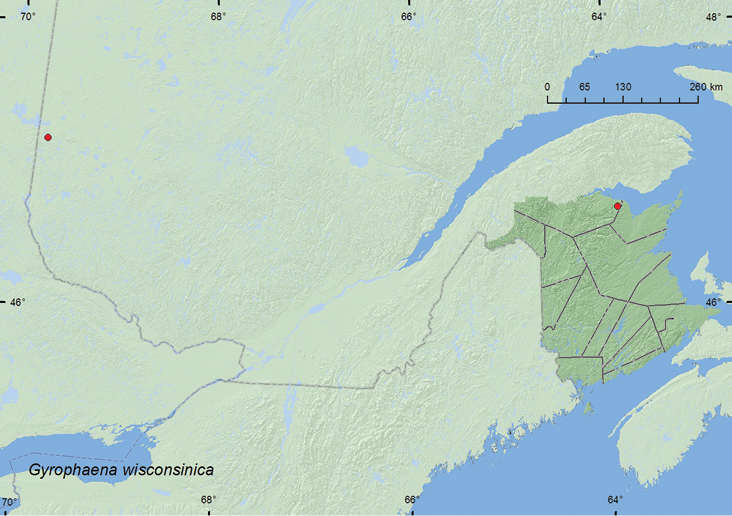
Collection localities in New Brunswick and Quebec, Canada of *Gyrophaena wisconsinica*.

Map 15 illustrations Seevers (1951)

###### Material examined.

**Canada, New Brunswick, Restigouche Co.**, Jacquet River Gorge P. N. A., 47.8201°N, 65.9992°W, 12.VIII.2010, R. P. Webster, black spruce/balsam fir/cedar forest near Belledune Bog, in gilled mushroom (1 ♂, RWC).

**Quebec**, Abitibi, lac Duparquet, (48.46926°N, 79.27164°W) 22.VIII.1997, Berlese, Éc. peup. mort + champ Échant. S-101 1944, Peupleraie, P. Paquin (20 ♂, 32 ♀ females, LFC)**.**

###### Collection and habitat data.

One individual of this species from New Brunswick was collected during August from a fresh gilled mushroom in a black spruce, balsam fir, and eastern white cedar forest. Specimens from Quebec were collected by Berlese extaction of dead poplar bark and mushrooms from a poplar forest. Samples were collected during late August.

###### Distribution in Canada and Alaska.

QC, **NB.** (new Canadian record) Seevers (1951) reported this species from Wisconsin and Illinois in the United States.

##### Leptusa gatineauensis

Klimaszewski & Pelletier, 2004

http://species-id.net/wiki/Leptusa_gatineauensis

**Map 16.**
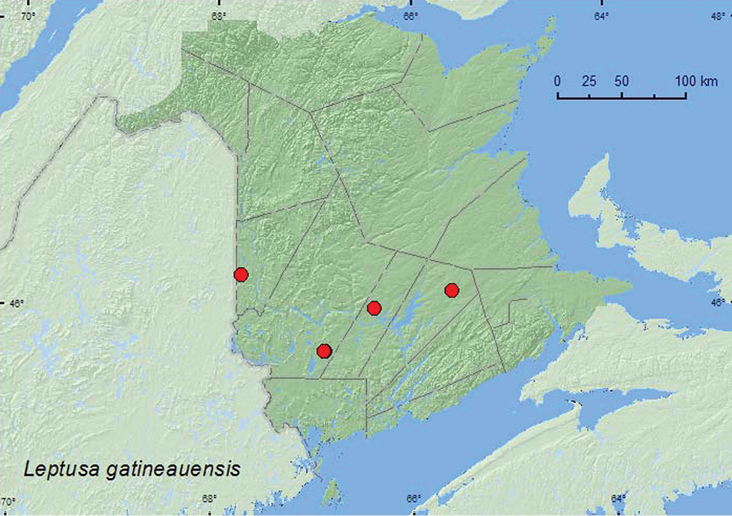
Collection localities in New Brunswick, Canada of *Leptusa gatineauensis*.

Map 16 illustrations in Klimaszewski et al. (2004)

###### Material examined.

**New Brunswick, Carleton Co.**, Jackson Falls, “Bell Forest”, 46.2200°N, 67.7230°W, 20-26.V.2009, R. Webster & M.-A. Giguère, rich Appalachian hardwood forest, Lindgren funnel trap (1 ♂, RWC). **Queens Co.**, Cranberry Lake P.N.A., 46.1125°N, 65.6075°W, 13-25.V.2011, 25.V–7.VI.2011, M. Roy & V. Webster, old red oak forest, Lindgren funnel traps (3 ♂, RWC). **Sunbury Co.**, Acadia Research Forest, 45.9866°N, 66.3841°W, 9-16.VI.2009, M.-A. Giguère & R. Webster mature (110-year-old) red spruce forest with scattered red maple and balsam fir, Lindgren funnel trap (1 ♀, RWC). **York Co.,** 15 km W of Tracy, off Rt. 645, 45.6848°N, 66.8821°W, 10-26.V.2010, 4–16.VI.2010, R. Webster & C. MacKay, coll., old red pine forest, Lindgren funnel traps (1 ♂, 2 ♀, LFC, RWC); 16 km W of Tracy, off Rt. 645, 45.6855°N, 66.8847°W, 18.V-2.VI.2010, R. Webster & C. MacKay, coll., old red pine forest, Lindgren funnel trap (1 ♂, RWC).

###### Collection and habitat data.

The specimens from New Brunswick were captured in Lindgren funnel traps deployed in an old red pine forest, a mature (110-year-old) red spruce forest, an old red oak forest, and a rich Appalachian hardwood forest. Elsewhere, adults were captured in deciduous and mature red spruce–hemlock forests; some specimens were captured on *Polyporus betulinus* (Bull.) Fries) (Klimaszewski et al. 2004). Adults were collected during May and June in both New Brunswick and Stanley Park, Vancouver, British Columbia (McLean et al. 2009).

###### Distribution in Canada and Alaska.

BC, ON, QC, **NB,** NS,NL (Klimaszewski et al. 2004; Gouix and Klimaszewski 2007; McLean et al. 2009).

##### Leptusa (Boreoleptusa) canonica

Casey, 1906

http://species-id.net/wiki/Leptusa_canonica

**Map 17.**
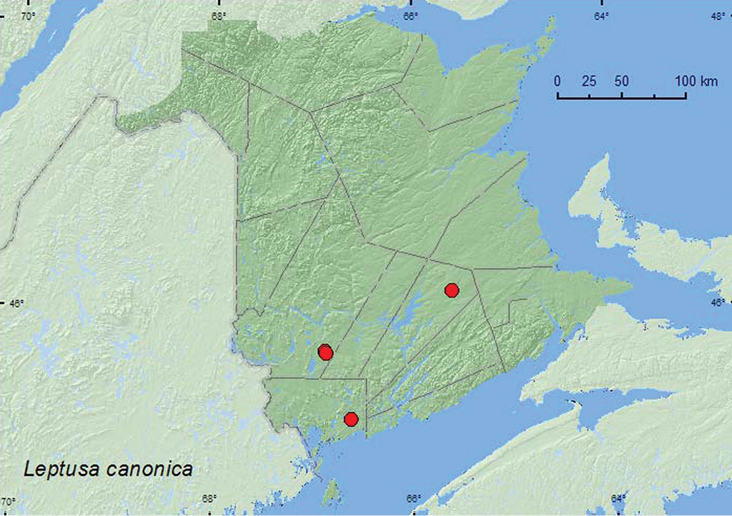
Collection localities in New Brunswick, Canada of *Leptusa canonica*.

Map 17 illustrations Klimaszewski et al. (2004)

###### Material examined.

**New Brunswick, Charlotte Co.**, 10 km NW of New River Beach, 45.2110°N, 66.6170°W, 10-23.VIII.2010, R. Webster and C. MacKay, old growth eastern white cedar forest, Lindgren funnel trap (1 sex undetermined, AFC). **Queens Co.**, Cranberry Lake P.N.A., 46.1125°N, 65.6075°W, 28.VI–1.VII.2009, 15-21.VII.2009, 21-28.VII.2009, 14–19.VIII.2009, 19.VIII-2.IX.2009, R. Webster and M.-A. Giguère, red oak forest, Lindgren funnel traps (5 ♂, 5 ♀, RWC). **York Co.**, 15 km W of Tracy off Rt. 645, 45.6848°N, 66.8821°W, 14–20.VII.2009, 20-29.VII.2009, R. Webster and M.-A. Giguère, old red pine forest, Lindgren funnel trap (1 sex undetermined, AFC); 14 km WSW of Tracy, S of Rt. 645, 45.6741°N, 66.8661°W, 16–30.VI.2010, R. Webster and C. MacKay, old mixed forest with red and white spruce, red and white pine, balsam fir, eastern white cedar, red maple, and *Populus* sp., Lindgren funnel trap (1 ♂, AFC).

###### Collection and habitat data.

Klimaszewski et al. (2004) reported this species from Lindgren funnel traps and four-winged intercept traps. Adults were collected in a yellow birch (*Betula alleghaniensis* Britt.)– balsam fir forest and an old-growth red spruce forest. In New Brunswick, this species was captured in Lindgren funnel traps deployed in an old red oak forest, an old mixed forest, an old red pine forest, and in an old-growth eastern white cedar forest. Adults were collected during June, July, and August.

###### Distribution in Canada and Alaska.

ON, QC, **NB**, NS, NF (Klimaszewski et al. 2004, 2011; Gouix and Klimaszewski 2007; Majka and Klimasewski 2010).

##### Silusa langori

Klimaszewski, 2003**

http://species-id.net/wiki/Silusa_langori

**Map 18.**
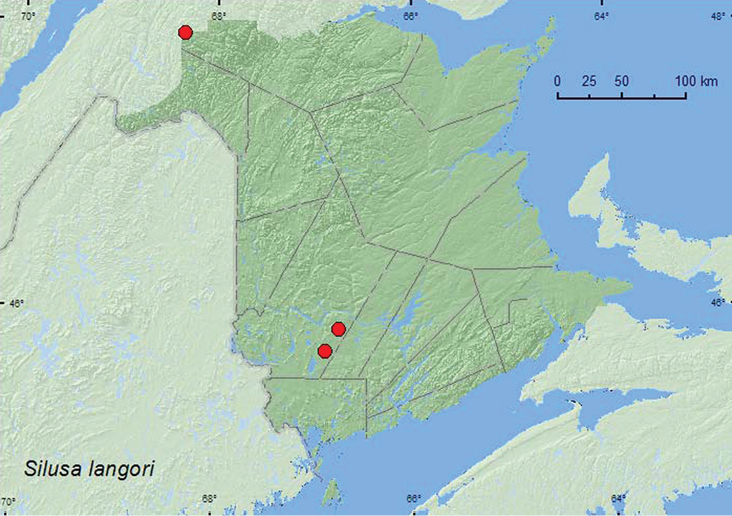
Collection localities in New Brunswick, Canada of *Silusa langori*.

Map 18 illustrations Klimaszewski et al. (2003)

###### Material examined.

**New Brunswick, Restigouche, Co.**, Dionne Brook P.N.A, 47.9064°N, 68.3441°W, 27.VI–14.VII.2011, M. Roy & V. Webster, old-growth white spruce and balsam fir forest, Lindgren funnel trap (1 ♂, RWC). **York Co.**, 15 km W of Tracy off Rt. 645, 45.6848°N, 66.8821°W, 19–25.V.2009, R. Webster and M.-A. Giguère, old red pine forest, Lindgren funnel trap (1 ♂, RWC); same locality data and forest type, 10–26.V.2010, R. Webster & C. MacKay, Lindgren funnel trap (1 ♂, RWC); Charters Settlement, 45.8395°N, 66.7391°W, 4.IV.2010, R. P. Webster, mixed forest opening, collected with aerial net during evening flight between 16:30 and 19:00 h (1 ♂, RWC).

###### Collection and habitat data.

In New Brunswick, adults of this species were collected during April, May, June, and July in Lindgren funnel traps deployed in an old red pine forest, an old-growth white spruce and balsam fir forest, and with an aerial net during an evening flight within a mixed forest opening. In Alberta, adults were captured in pitfall traps and window traps in boreal mixed woods comprising 54–83% *Populus* sp.(Klimaszewski et al. 2003).

###### Distribution in Canada and Alaska.

AB, **NB** (Klimaszewski et al. 2003). The New Brunswick records represent a significant eastward range extension for this species.

#### Tribe Athetini Casey, 1910

##### Acrotona smithi

Casey, 1910**

http://species-id.net/wiki/Acrotona_smithi

**Map 19.**
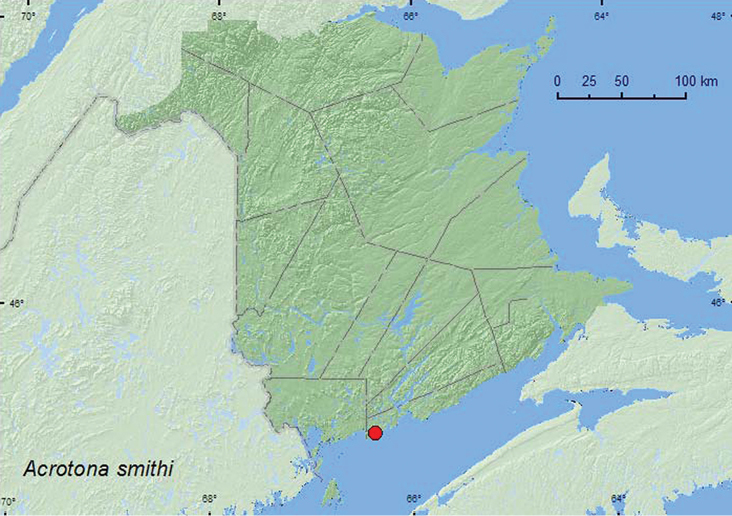
Collection localities in New Brunswick, Canada of *Acrotona smithi*.

Map 19 illustrations in Brunke et al. (2012)

###### Material examined.

**New Brunswick, Saint John Co.**, Dipper Harbour, 45.1169°N, 66.3771°W, 7.V.2006, 15.V.2006, 30.V.2006, R. P. Webster, upper margin sea beach, in decaying sea wrack and debris under alders (2 ♂, 1 ♀, 1 sex undetermined, LFC; 3 ♂, 4 ♀, 1 sex undetermined, RWC); same locality and collector but 45.1154°N, 66.3720°W, 12.V.2008, sea beach, in decaying sea wrack on gravel and sand (1 ♂, 1 ♀, 1 sex undetermined, RWC.

###### Collection and habitat data.

Specimens of *Acrotona smithi* Casey from New Brunswick were collected during May on the upper margin of a sea beach from decaying sea wrack under alders (*Alnus* sp.) and on gravel and sand.

###### Distribution in Canada and Alaska.

**NB.** This species is more widely distributed in eastern Canada, and all other new records are reported in Brunke et al. (2012).

##### Acrotona sequestralis

(Casey, 1910)**

http://species-id.net/wiki/Acrotona_sequestralis

**Map 20.**
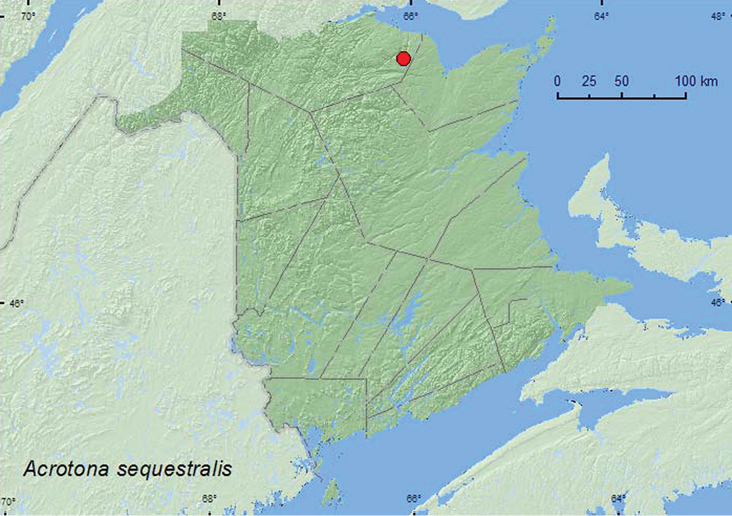
Collection localities in New Brunswick, Canada of *Acrotona sequestralis*.

Map 20 illustrations in Klimaszewski et al. (2011)

###### Material examined.

**New Brunswick, Restigouuche Co.,** Jacquet River Gorge P.N.A., 47.7361°N, 66.0778°W, 16.VIII.2010, R. P. Webster, coll., beaver dam, among sticks and debris near an overflow area of dam (near flowing water) (1 ♂, RWC).

###### Collection and habitat data.

Specimens of *Acrotona sequestralis* (Casey) from New Brunswick were collected in August from debris near an overflow area of a beaver dam. Klimaszewski et al. (2011) reported this species in June from along the shoreline of an inlet containing brackish water.

###### Distribution in Canada and Alaska.

NL, **NB** (Klimaszewski et al. 2011). Presently, this species is known only from the above two provinces but most likely is more widely distributed in eastern Canada.

##### Atheta (Atheta) circulicollis

Lohse, 1990**

http://species-id.net/wiki/Atheta_circulicollis

**Map 21.**
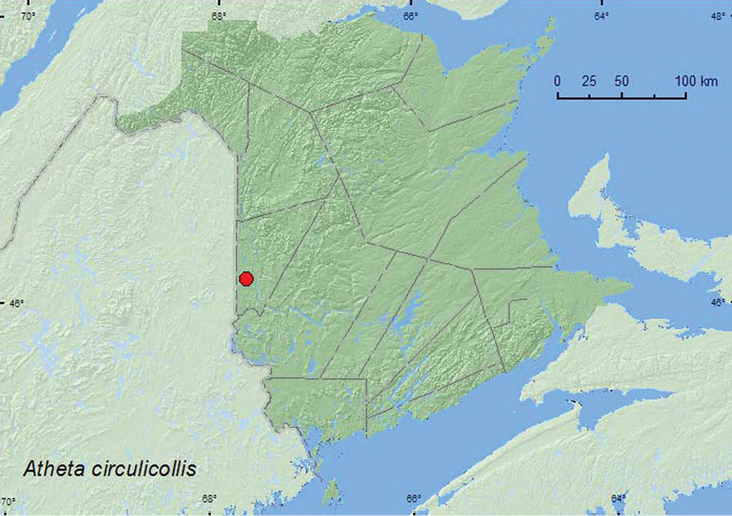
Collection localities in New Brunswick, Canada of *Atheta circulicollis*.

Map 21 illustrations Klimaszewski et al. (2011)

###### Material examined.

**New Brunswick, Carleton Co.**, Belleville, Meduxnekeag Valley Nature Preserve, 46.1910°N, 67.6740°W, 13.VIII.2006, R. P. Webster, mixed forest, on *Pleurotus* sp. on side of log (1 ♂, RWC).

###### Collection and habitat data.

The only specimen of this species from New Brunswick was collected during August from a *Pleurotus* sp. on the side of a log. Specimens from NF & LB were captured in pitfall traps deployed in fir forests, riparian forests, and a recently burned coniferous forest (Klimaszewski et al. 2011).

###### Distribution in Canada and Alaska.

QC, **NB**, NF, LB (Lohse et al. 1990; Klimaszewski et al. 2011).

##### Atheta (Dimetrota) pseudomodesta

Klimaszewski, 2007

http://species-id.net/wiki/Atheta_pseudomodesta

**Map 22.**
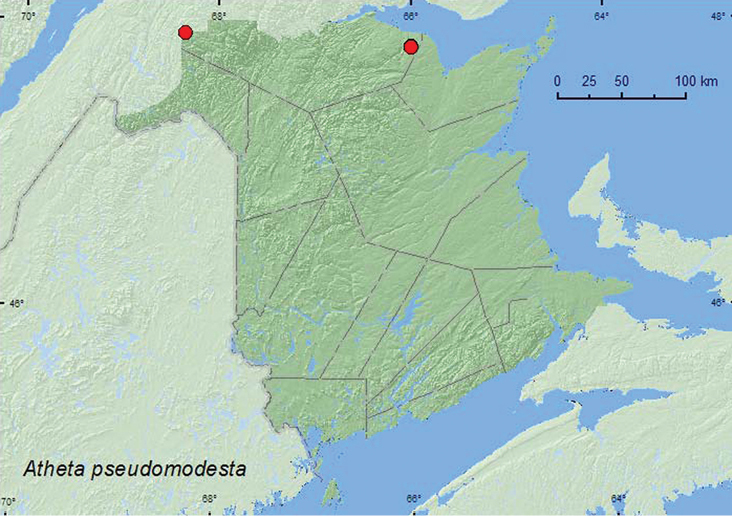
Collection localities in New Brunswick, Canada of *Atheta pseudomodesta*.

Map 22 illustrations Klimaszewski et al. (2007b)

###### Material examined.

**New Brunswick, Restigouche Co.** Jacquet River Gorge P.N.A., 47.8207°N, 65.9955°W, 15.VI.2009, R.P. Webster, black spruce forest with *Populus* sp., on gilled mushroom (1 ♂, 1 ♀, NBM, RWC); same locality and collector but 47.8201°N, 65.9992°W, 12.VIII.2010, black spruce/balsam fir/cedar forest near Belledune Bog, in gilled mushroom (1 ♂, NBM); Dionne Brook P.N.A., 47.9030°N, 68.3503°W, 30.V–15.VI.2011, M. Roy & V. Webster, old-growth northern hardwood forest, Lindgren funnel trap (1, RWC).

###### Collection and habitat data.

Klimaszewski et al. (2007b) reported this species as abundant in yellow birch forests in Quebec. The specimens from New Brunswick were collected from gilled mushrooms in a black spruce forest with *Populus* sp. and in a black spruce, balsam fir, and eastern white cedar forest. One individual was captured in a Lindgren funnel trap deployed in an old-growth, northern hardwood forest. Adults were collected during May, June, and August in New Brunswick.

###### Distribution in Canada and Alaska.

ON,QC, **NB,** NS, NL (Klimaszewski et al. 2007b, 2011; Majka and Klimaszewski 2008).

##### Atheta (Dimetrota) terranovae

Klimaszewski & Langor, 2011**

http://species-id.net/wiki/Atheta_terranovae

**Map 23.**
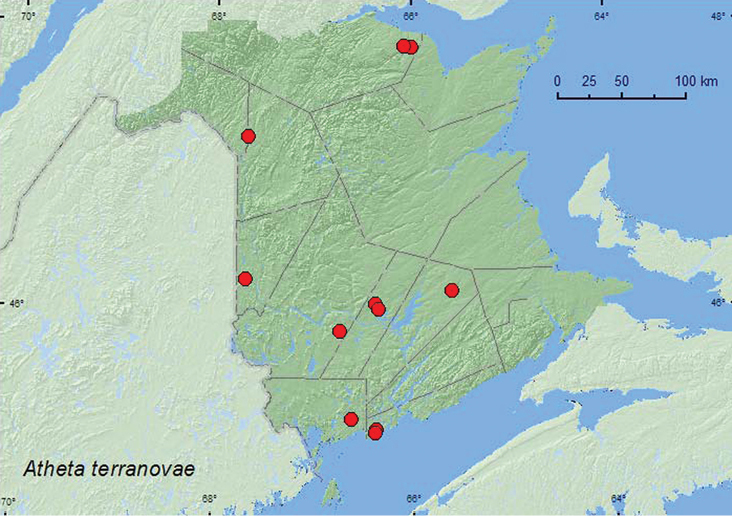
Collection localities in New Brunswick, Canada of *Atheta terranovae*.

Map 23 illustrations in Klimaszewski et al. (2011)

###### Material examined.

**New Brunswick, Carleton Co.**, Belleville, Meduxnekeag Valley Nature Preserve, 46.1927°N, 67.6803°W, 4.V.2006, R. P. Webster, 16.IX.2006, R. P. Webster, coll., mixed forest in decaying gilled mushrooms (1 ♂, RWC; 1 ♂, LFC); same locality and collector except, 46.1907°N, 67.6740°W, 14.IX.2005, mixed forest on gilled fungi (1 ♂, 1 ♀, RWC; 1 ♂, LFC); same locality data and collector, 7.IX.2004, mixed forest on rotting fungi (1 ♀, RWC). **Charlotte Co.**, near New River, 45.2122°N, 66.6160°W, 22.IX.2006, R. P. Webster, coll., eastern white cedar swamp, in gilled mushroom (1 ♂, RWC). **Queens Co.**, Cranberry Lake P.N.A., 46.1125°N, 65.6075°W, 22.IX.2009, R. P. Webster, coll., red oak forest, in decaying gilled mushrooms (1 ♂, RWC ). **Restigouche Co.**, Jacquet River Gorge P.N.A., 47.8201°N, 65.9992°W, 12.VIII.2010, R. P. Webster, coll., black spruce forest, in gilled mushrooms (1 ♀, NBM); same locality and collector but 47.8254°N, 66.0780°W, 18.VIII.2010, spruce /fir forest, in decaying lobster mushrooms (1 ♂, RWC ). **Saint John Co.**, Chance Harbour (off Rt. 790), 45.1391°N, 66.3696°W, 24.VIII.2006, R. P. Webster, coll., red spruce & birch forest, in gilled mushrooms (1 ♂, 1 ♀, RWC); same locality data and collector, 16.IX.2008, mixed forest, in decaying gilled mushrooms (1 ♂, NBM); Dipper Harbour, 45.1176°N, 66.3806°W, 12.IX.2006, R. P. Webster, coll., red spruce forest, on gilled mushrooms (1 ♀, RWC). **Sunbury Co.**, Acadia Research Forest, 46.0188°N, 66.3765°W, 18.IX.2007, R. P. Webster, coll., Road 16 control, mature red spruce & red maple forest, in coral fungi on spruce log (1 ♂, 1 ♀, RWC); same locality and collector but 45.9799°N, 66.3394°W, 18.IX.2007, Road 7 control, mature red spruce & red maple forest, in gilled mushrooms (1 ♂, RWC). **York Co**., Charters Settlement, 45.8286°N, 66.7365°W, 6.IX.2005, 4.X.2005, R. P. Webster, coll., mature red spruce & cedar forest, in decaying mushrooms (3 ♀, RWC).

###### Collection and habitat data.

Klimaszewski et al. (2011) reported this species from coniferous, mixed, and deciduous forests in NF & LB. Specimens were captured in carrion-baited pitfall traps, unbaited pitfall traps, and flight intercept traps during June, July, and August. Some adults were collected from decaying mushrooms in forests (Klimaszewski et al. 2011). Most specimens from New Brunswick were collected from fresh and decaying gilled mushrooms. One individual was collected from a rotting lobster mushroom and another from a coral mushroom on a spruce log. This species was found in mixed forests, mature red spruce forests with red maple or birch, a black spruce forest, an eastern white cedar swamp, and a red oak forest. Adults from New Brunswick were collected during August, September (most specimens), and October.

###### Distribution in Canada and Alaska.

**NB,** NL, QC (Klimaszewski et al. 2011). This species is probably more widely distributed in eastern Canada.

##### Atheta (Microdota) pseudosubtilis

Klimaszewski & Langor, 2011**

http://species-id.net/wiki/Atheta_pseudosubtilis

**Map 24.**
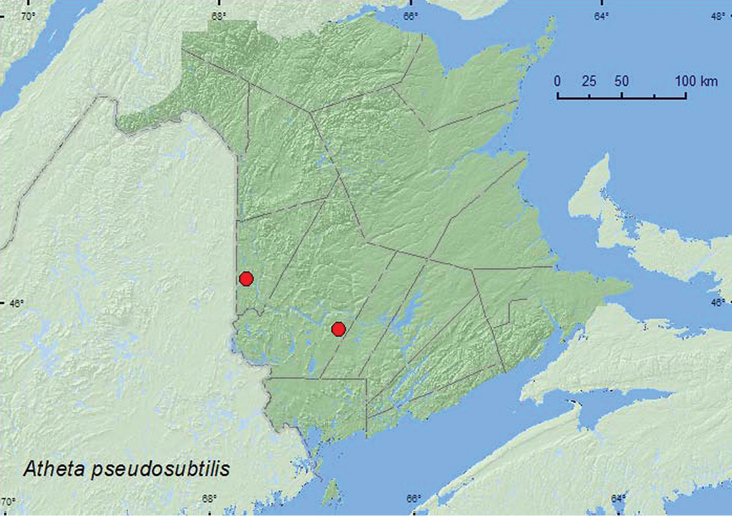
Collection localities in New Brunswick, Canada of *Atheta pseudosubtilis*.

Map 24 illustrations in Klimaszewski et al. (2011)

###### Material examined.

**New Brunswick, Carleton Co.,** Belleville, Meduxnekeag Valley Nature Preserve, 46.1910°N, 67.6740°W, 4.V.2006, R. P. Webster, balsam fir stand, in moldy conifer duff at base of white pine,(4 ♂, 2 ♀, RWC; 1 ♂, LFC); same locality data and collector except 46.1907°N, 67.6740°W, 11.V.2005, balsam fir stand, in moldy conifer duff (2 ♀, RWC). **York Co**., Charters Settlement, 45.8395°N, 66.7391°W, 22.IV.2004, R. P. Webster, coll., mixed forest, in leaf litter & moss near small shaded stream (1 ♀, RWC).

###### Collection and habitat data.

Specimens from New Brunswick were collected from moldy conifer duff at the base of a white pine in a balsam fir stand during May and from leaf litter and moss near a small, shaded stream in a mixed forest during April. The Newfoundland specimens were captured from June through August in mixed wood and coniferous forests, using unbaited and carrion-baited pitfall and intercept traps (Klimaszewski et al. 2011).

###### Distribution in Canada and Alaska.

**NB,** NL (Klimaszewski et al. 2011).

##### Clusiota impressicollis

(Bernhauer, 1907)**

http://species-id.net/wiki/Clusiota_impressicollis

**Map 25.**
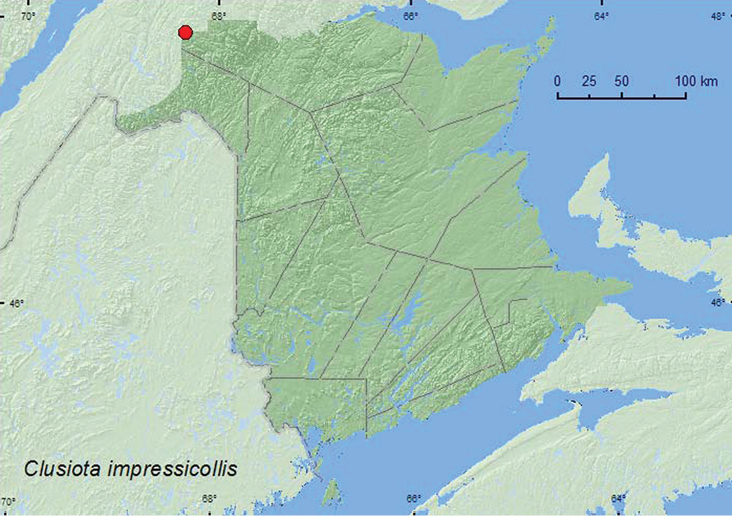
Collection localities in New Brunswick, Canada of *Clusiota impressicollis*

Map 25 illustrations in Klimaszewski et al. (2011)

###### Material examined.

**New Brunswick, Restigouche Co.,** Dionne Brook P.N.A., 47.9064°N, 68.3441°W, R. P. Webster, 9.VIII.2011, under coyote dung on gravel road (1 ♂, RWC).

###### Collection and habitat data.

The specimen from New Brunswick was collected from under coyote (*Canis latrans* Say) dung on a gravel road during August. Specimens from Newfoundland were collected in flight intercept traps deployed in a fir–decidous forest during July–August. Otherwise little is known about the habitat requirements and biology of this species.

###### Distribution in Canada and Alaska.

BC, ON, **NB,** NL (Gouix and Klimaszewski 2007; Majka and Klimaszewski 2008; Klimaszewski et al. 2011).

##### Hydrosmecta pseudodiosica

Lohse, 1990**

http://species-id.net/wiki/Hydrosmecta_pseudodiosica

**Map 26.**
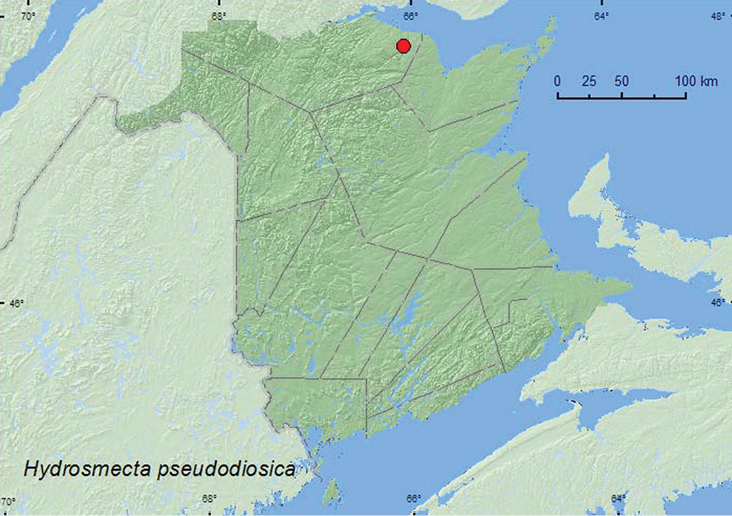
Collection localities in New Brunswick, Canada of *Hydrosmecta pseudodiosica*.

Map 26 illustrations in Lohse et al. (1990)

###### Material examined. 

**New Brunswick, Restigouche Co.,** Jacquet River Gorge P.N.A., 47.8257°N, 66.0779°W, 14.V.2010, R. P. Webster, coll., partially shaded cobblestone bar near outflow of brook at the Jacquet River, under cobblestones and gravel on sand (3 ♂, 1 ♀, RWC; 1 ♂, LFC); same locality and habitat data and collector except 24.V.2010 (4 ♂, 3 ♀, RWC); same locality and collector but 47.8257°N, 66.0768°W, 16.VI.2009, balsam poplar forest, medium sized stream near outflow into Jacquet River, on partially shaded cobblestone island, among cobblestones (2 ♀, RWC).

###### Collection and habitat data.

The specimens from New Brunswick were collected from under small cobblestones and gravel set in sand on a partially shaded cobblestone bar near the outflow of a brook into a clear rocky river. Adults were collected during May and June. Lohse et al. (1990) recorded this species from edges of running water in Yukon.

###### Distribution in Canada and Alaska.

YT, ON, **NB,** (Lohse et al. 1990; Majka and Klimaszewski 2008).

##### Hydrosmecta newfoundlandica

Klimaszewski & Langor, 2011**

http://species-id.net/wiki/Hydrosmecta_newfoundlandica

**Map 27.**
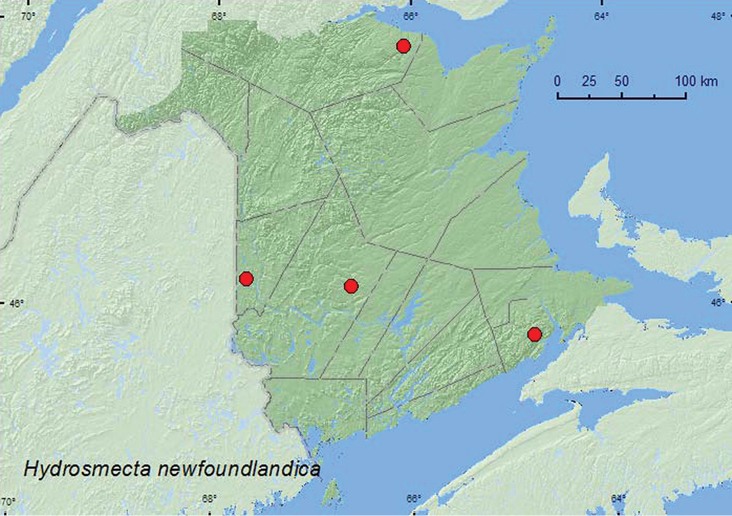
Collection localities in New Brunswick, Canada of *Hydrosmecta newfoundlandica*.

Map 27 illustrations in Klimaszewski et al. (2011)

###### Material examined.

**New Brunswick, Albert Co.,** Caledonia Gorge P.N.A., at Crooked Creek, 45.7930°N, 64.7764°W, 1.VII.2011, R. P. Webster, small clear cold rocky river, among cobblestones on river margin (1 ♂, NBM). **Carleton Co.,** Belleville, Meduxnekeag Valley Nature Preserve, 46.1897°N, 67.6751°W, 19.VII.2009, R. P. Webster, rich Appalachian hardwood forest, margin of spring-fed brook among gravel on firm sand/clay/gravel mix (1 ♂, RWC). **Restigouuche Co.,** Jacquet River Gorge P.N.A., 47.8257°N, 66.0768°W, 16.VI.2009, 14.V.2010, R. P. Webster, coll., balsam poplar forest, medium sized stream near outflow into Jacquet River, on partially shaded cobblestone island among cobblestones, (4 ♂, 3 ♀, 1 sex undetermined, RWC, LFC). **York Co.,** 1.5 km N of Durham Bridge, 46.1408°N, 66.6179°W, 15.VI.2008, R. P. Webster, coll., Nashwaak River at river margin among cobblestones near outflow of a brook, (7 ♂, 4 ♀, RWC, LFC).

###### Collection and habitat data.

Most specimens from New Brunswick were collected from among cobblestones along clear, rocky river margins near the outflow of brooks. One individual was collected from the margin of a spring-fed brook among gravel on firm sand/clay/gravel mix near the outflow of the brook into a clear, rocky river. Specimens were usually found among cobblestones at waters edge. Adults were collected during May, June, and July. Adults from Newfoundland were captured from an unknown habitat in July and August (Klimaszewski et al. 2011).

###### Distribution in Canada and Alaska.

**NB**,NL (Klimaszewski et al. 2011).

##### Tomoglossa decora

(Casey, 1910 )***

http://species-id.net/wiki/Tomoglossa_decora

**Map 28.**
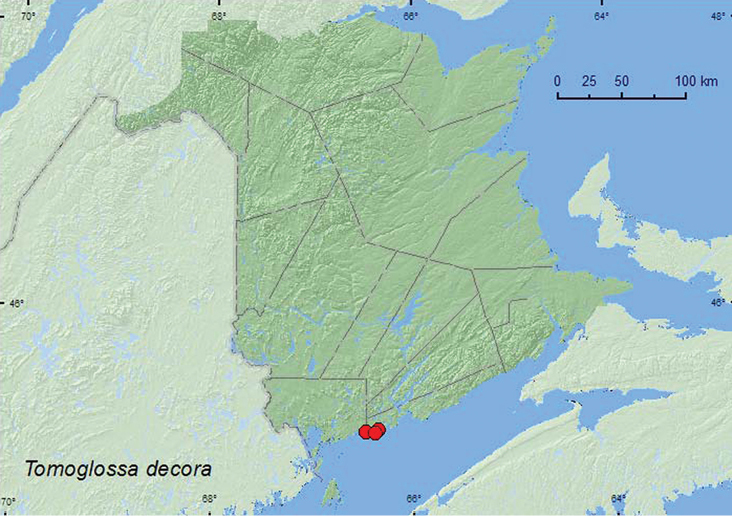
Collection localities in New Brunswick, Canada of *Tomoglossa decora*.

**Figure 5.**
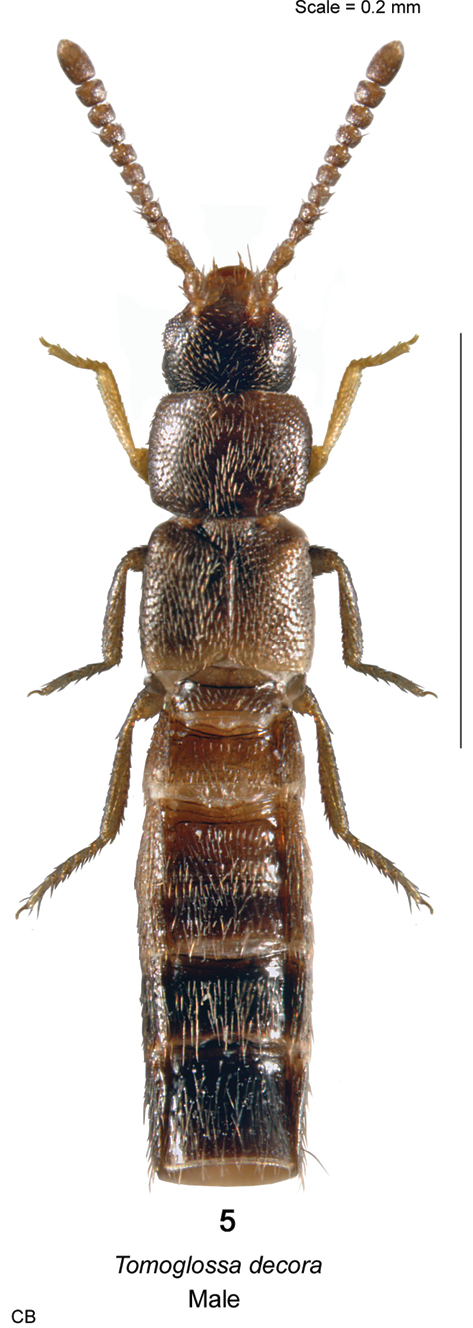
*Tomoglossa decora* (Casey) habitus in dorsal view.

Fig. 5 Map 28 illustrations in Gusarov (2002)

###### Material examined.

**Canada, New Brunswick, Charlotte Co.**, near Little Lepreau, 45.1242°N, 66.4732°W, 11.VII.2008, R. P. Webster, coll., barrier beach, intertidal zone, under small rocks in sand/clay mix near small bay, 1 to 2 meters below mean high tide mark (2 ♂, RWC ). **Saint John Co.,** Chance Harbour off Cranberry Head Rd., 45.1350°N, 66.3439°W, 6.VII.2008, R. P. Webster coll., barrier beach, intertidal zone, under cobble stones in sand adjacent to salt marsh, about 0.5 meters below mean high tide mark, (2 ♀, RWC); same locality and collector except 45.1357°N, 66.3451°W, 11.VII.2008, under cobble stones in sand adjacent to salt marsh, about 0.5-2.0 meters below mean high tide mark (1 ♂, LFC; 1 ♀, RWC ); same locality and collector but 45.1354°N, 66.3438°W, salt marsh, under small rock on salt marsh side of barrier beach (1 ♂, RWC ); Chance Harbour, 45.1173°N, 66.3766°W, 25.VI.2010, R. P. Webster, salt marsh adjacent to barrier beach, under small rock among *Spartina patens* (1 ♀, RWC).

###### Collection and habitat data.

In New Brunswick, this species was found on the salt marsh side of barrier beaches in the intertidal zone, 1–2 m below the mean high-tide mark. Adults occurred under small rocks set in sand or a sand–clay mix in areas with sparse *Spartina patens* (Ait.) Muhl. (salt meadow grass). Adults were collected during June and July.

###### Distribution in Canada and Alaska.

**NB** (new Canadian record). Gusarov (2002) reported this species from the eastern USA.

##### Liogluta aloconotoides

Lohse, 1990

http://species-id.net/wiki/Liogluta_aloconotoides

**Map 29.**
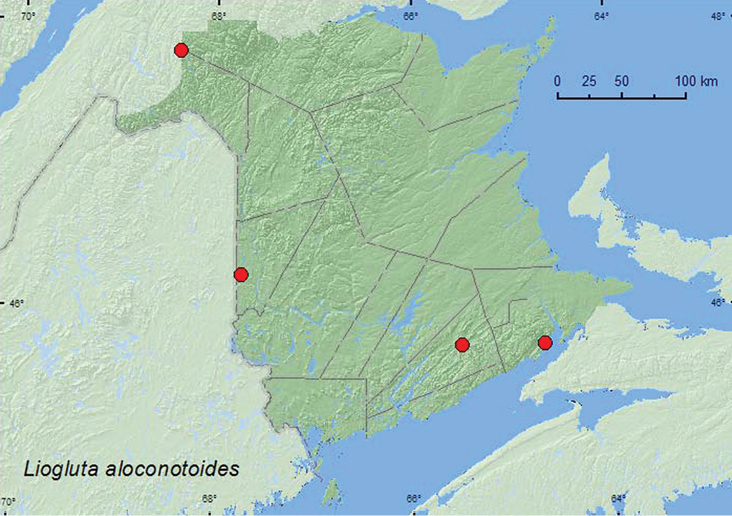
Collection localities in New Brunswick, Canada of *Liogluta aloconotoides*.

Map 29 illustrations in Klimaszewski et al. (2011)

###### Material examined.

**New Brunswick, Albert Co.**, Shepody N.W.A., Mary’s Point Section, 45.7320°N, 64.6765°W, 12.IX.2004, R. P. Webster, spruce forest, in dung (1 ♀, RWC). **Carleton Co.**, Jackson Falls, “Bell Forest”, 46.2200°N, 67.7231°W, 12-19.VII.2008, R. P. Webster, rich Appalachian hardwood forest with some conifers, Lindgren funnel trap (1 ♂, RWC). **Kings Co.**, Sussex, 3.VIII.1994 (J. Sweeney), pitfall control 3-3 (1 ♀, AFC). **Madawaska Co.**, Loon Lake, 236 m elev., 47.7839°N, 68.3843°W, 21.VII.2010, R. P. Webster, boreal forest, small lake surrounded by sedges, treading sedges near *Myrica gale* bushes (1 ♂, 1 ♀, RWC).

###### Collection and habitat data. 

Klimaszewski et al. (2011) reported this species from various forest types and on coastal limestone barrens in Newfoundland. Specimens from New Brunswick were collected from dung in a coastal red spruce forest, treading sedges along a small lake margin, from a Lindgren funnel trap deployed in a rich Appalachian hardwood forest with some conifers, and in a pitfall trap. Adults were collected during July, August, and September.

###### Distribution in Canada and Alaska.

YT, ON, QC, **NB**, NS, LB, NF (Lohse et al. 1990; Majka and Klimaszewski 2008; Klimaszewski et al. 2008a).

##### Lypoglossa angularis obtusa

(LeConte, 1866)

http://species-id.net/wiki/Lypoglossa_angularis_obtusa

**Map 30.**
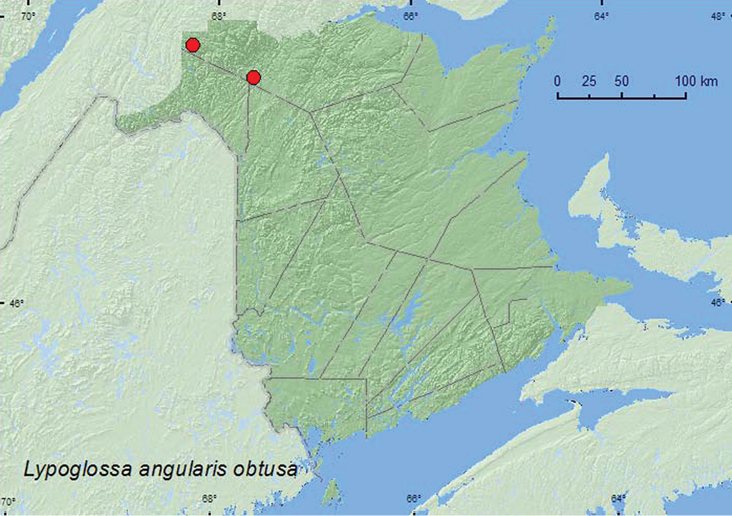
Collection localities in New Brunswick, Canada of *Lypoglossa angularis obtusa*.

Map 30 illustrations in Gusarov (2004) Klimaszewski et al. (2011)

###### Material examined.

**New Brunswick, Restigouche Co.**, MacFarlane Brook Protected (Natural) Area, 47.6018°N, 67.6263°W, 25.V.2007, R. P. Webster, old growth eastern white cedar swamp, in moss and leaves under alders near brook (1 ♀, RWC); Mount Atkinson, 447 m elev., 47.8192°N, 68.2618°W, 24.VIII.2011, R. P. Webster, spruce and balsam fir forest, small, shaded, spring-fed brook with mossy margin, in wet moss (1 sex undetermined, RWC).

###### Collection and habitat data.

In Newfoundland,this species has been captured in unbaited and carrion-baited pitfall traps in old balsam fir, spruce and balsam fir, birch and riparian forests and shrubby coastal barrens (Klimaszewski et al. 2011). Gusarov (2004) reported this species from *Abies*, *Betula*, *Picea*, and *Oxalis* litter. The specimens from Nova Scotia were captured in a pan trap in a “closed” spruce woodland (Majka and Klimaszewski 2010). Specimens from New Brunswick were sifted from moss and leaves under alders near a brook in an old-growth eastern white cedar swamp and from wet moss on the margin of a small, shaded, spring-fed brook in a white spruce and balsam fir forest. The adults were collected during May and August. Elsewhere, this species has been collected from June to October.

###### Distribution in Canada and Alaska.

QC, **NB**, NS, LB, NF (Gusarov 2004; Majka and Klimaszewski 2010; Klimaszewski et al. 2011). Makja and Klimaszewski (2010) reported this species for the first time from Nova Scotia on the basis of a specimen collected in Louisburg, Cape Breton Co.

##### Philhygra jarmilae

Klimaszewski & Langor, 2011**

http://species-id.net/wiki/Philhygra_jarmilae

**Map 31.**
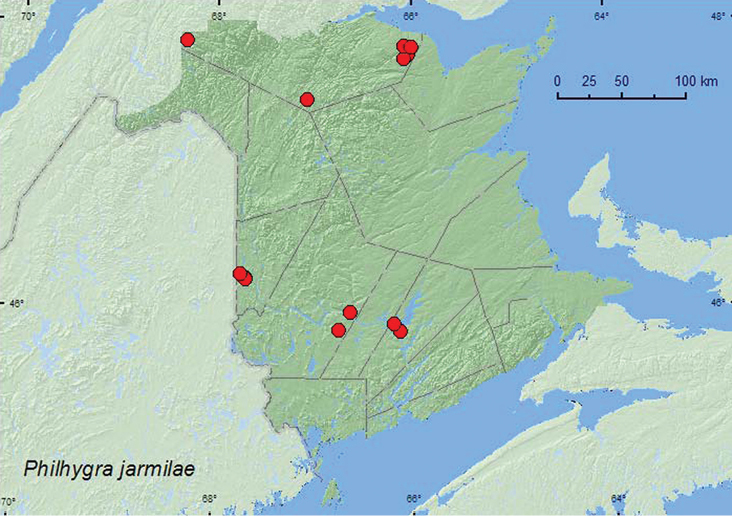
Collection localities in New Brunswick, Canada of *Philhygra jarmilae*.

Map 31 illustrations Klimaszewski et al. (2011)

###### Material examined.

**New Brunswick, Carleton Co.**, (Belleville) Meduxnekeag Valley Nature Preserve, 46.1976°N, 67.6850°W, 4.V.2006, R. P. Webster, mixed forest, margin of vernal pond, in moist leaf litter (1 ♀, RWC); 1.3 km E jct. Rt. 540 & Plymouth Rd., 46.1867°N, 67.6817°W, 7.V.2008, R. P. Webster, rich Appalachian hardwood forest, in moss & leaf litter in seepage area (1 ♀, RWC); Jackson Falls, 46.2257°N, 67.7437°W, 12.IX.2009, R. P. Webster, river margin near waterfall, splashing moss near splash zone of waterfall (1 ♂, RWC). **Queens Co.**, W of Jemseg near “Trout Creek”, 45.8255°N, 66.1174°W, 1.VII.2008, R. P. Webster, seasonally flooded marsh, treading vegetation near margin of pool (1 ♂, 1 ♀, RWC); Canning, Grand Lake near Scotchtown, 45.8762°N, 66.1817°W, 25.V.2006, R. P. Webster, silver maple swamp near lake margin, margin of vernal pond, in moist leaves (3 ♂, 1 ♀, NBM, RWC). **Restigouche Co.**, Little Tobique R. near Red Brook, 47.4465°N, 67.0689°W, 13.VI.2006, R. P. Webster, alder swamp near river, in debris on muddy soil near brook (1 ♂, RWC); Jacquet River Gorge P.N.A., 47.7627°N, 66.0270°W, 24.VI.2008, R. P. Webster, hardwood forest, margin of vernal pool, among moist leaves (1 ♀, RWC); same locality and collector but 47.7357°N, 66.0774°W, 24.VI.2008, among leaves and sedges near pond margin (1 ♂, RWC); same locality and collector but 47.8257°N, 66.0768°W, 16.VI.2009, balsam poplar forest, medium sized stream near outflow into Jacquet River, on partially shaded cobblestone island, among cobblestones (1 ♂, NBM); same locality and collector but 47.8200°N, 66.0015°W, 13.V.2010, under alders in leaf litter & moss near small brook in *Carex* marsh (1 ♂, NBM); Wild Goose Lake, 420 m elev., 47.8540°N, 68.3219°W, 7.VI.2011, R. P. Webster & M. Turgeon, lake margin with emergent *Carex* & grasses, treading *Carex* & grasses (1 ♂, NBM). **York Co.**, Fredericton, at St. John River, 45.9588°N, 66.6254°W, 4.VII.2004, R. P. Webster, margin of river, in drift material (mostly maple seeds) (1 ♂, NBM); Charters Settlement, 45.8340°N, 66.7450°W, 29.V.2008, R. P. Webster, mature mixed forest, margin of vernal pond, among moist leaves (1 ♂, RWC).

###### Collection and habitat data.

In New Brunswick, *Philhygra jarmilae* was found in various wetland habitats. Adults were sifted from moist leaves along the margin of vernal ponds in mixed forests, a hardwood forest, and a silver maple swamp, sifted from leaves and sedges along a pond margin, treaded from *Carex* and grasses along a lake margin, sifted from moss and leaf litter in a seepage area in a hardwood forest, treaded from vegetation in a seasonally flooded marsh near a pool, sifted from debris on muddy soil near a brook, sifted from leaf litter and moss under alders near a brook, and from drift material on a river margin, hand collected from cobblestones on a partially shaded cobblestone bar along a medium-sized stream, and collected by splashing water on moss near the splash zone of a waterfall. Adults were captured during May, June, July, and September in New Brunswick. The holotype was captured in a flight intercept trap in a mixed forest (Klimaszewski et al. (2011), otherwise nothing was previously known about the bionomics of this species.

###### Distribution in Canada and Alaska.

**NB**, NF (Klimaszewski et al. 2011).

##### Philhygra luridipennis

Mannerheim, 1831**

http://species-id.net/wiki/Philhygra_luridipennis

**Map 32.**
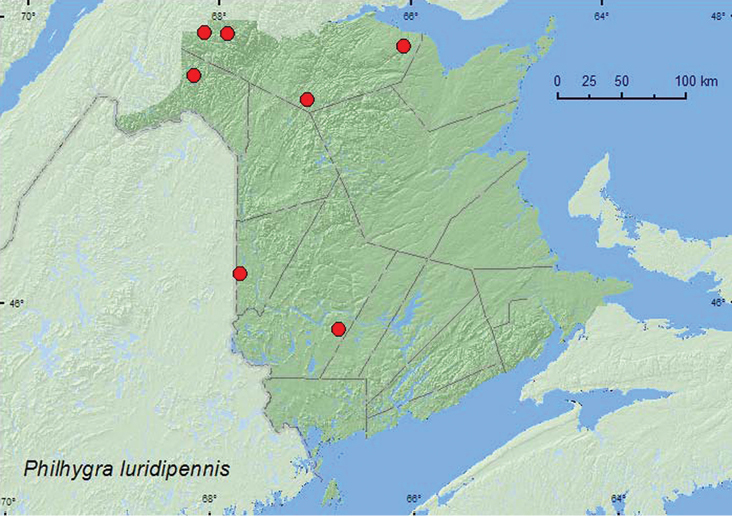
Collection localities in New Brunswick, Canada of *Philhygra luridipennis*.

Map 32 illustrations Klimaszewski et al. (2011)

###### Material examined.

**New Brunswick, Carleton Co.** Jackson Falls, 46.2257°N, 67.7437°W, 12.IX.2009, R. P. Webster, river margin near waterfall, splashing moss near splash zone of waterfall (1 ♀, RWC). **Madawaska Co.**, Gagné Brook at First Lake, 47.6077°N, 68.2534°W, 23.VI.2010, M. Turgeon & R. Webster, northern hardwood forest, shaded brook among gravel on gravel bar, splashing, turning gravel (1 ♂, RWC). **Restigouche Co.**, Little Tobique R. near Red Brook, 47.4465°N, 67.0689°W, 13.VI.2006, R. P. Webster, alder swamp near river, in debris on muddy soil near brook (1 ♀, RWC); Jacquet River Gorge P.N.A., 47.8257°N, 66.0768°W, 16.VI.2009, R. P. Webster, mixed mature forest, cool clear medium sized stream, in gravel & under cobble stones near margin of stream (1 ♂, 1 ♀, RWC); 1.5 km S of Quebec (border), 425 m elev., 47.9058°N, 68.1505°W, 22.VI.2010, R. P. Webster, boreal forest, small shaded brook, splashing gravel on gravel bar (1 ♂, RWC); Kedgwick Forks, 47.9085°N, 67.9057°W, 22.VI.2010, R. P. Webster, river margin on clay/sand, under alders (1 ♀, RWC). **York Co.**, Charters Settlement, 45.8395°N, 66.7391°W, 26.VII.2005, R. P. Webster, mixed forest, M.V. light (1 ♀, RWC); same locality data and collector, 21.IV.2010, mixed forest opening, collected with aerial net during evening flight between 16:30 and 19:00 h (1 ♂, RWC).

###### Collection and habitat data.

Most adults of *Philhygra luridipennis* from New Brunswick were collected from riparian habitats in various deciduous and coniferous forest types. Specimens were collected from gravel (splashing and turning gravel) on gravel bars along shaded brooks in a northern hardwood forest and a boreal forest with balsam fir and white spruce, hand collected from gravel and from under cobblestones on the margin of a clear, medium-sized stream in a mixed forest, sifted from debris on muddy soil near a brook in an alder swamp, hand collected from a sand and clay mix under alders near a river margin, and collected by splashing water on moss near the splash zone of a waterfall. Other specimens were collected at a mercury vapor light and with an aerial net during an evening flight near a mixed forest and nearby stream. Adults were collected during April, June, July, and September. Little was previously known about the habitat associations of this species. The male specimen from Newfoundland was captured in a flight intercept trap in a mixed forest (Klimaszewski et al. 2011).

###### Distribution in Canada and Alaska.

**NB,** NF (Klimaszewski et al. 2011). This species is either Holarctic or an adventive Palaearctic species in North America (Klimaszewski et al. 2011).

##### Philhygra sinuipennis

Klimaszewski & Langor, 2011**

http://species-id.net/wiki/Philhygra_sinuipennis

**Map 33.**
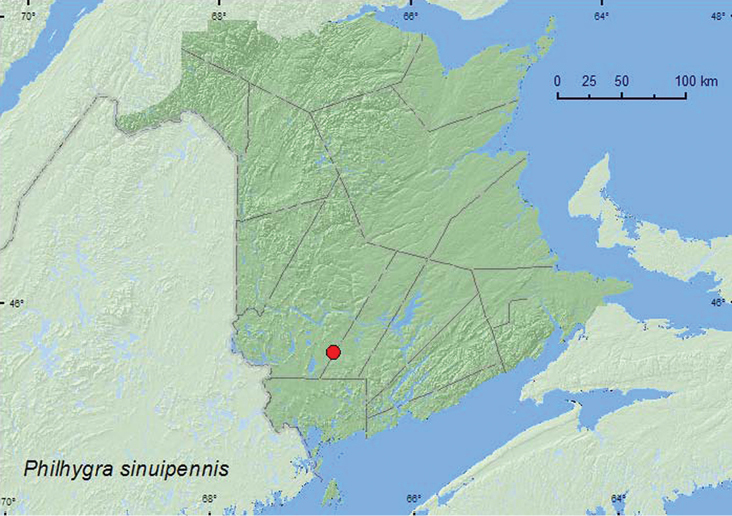
Collection localities in New Brunswick, Canada of *Philhygra sinuipennis*.

Map 33 illustrations Klimaszewski et al. (2011)

###### Material examined.

**New Brunswick, York Co.**8.5 km W of Tracy off Rt. 645, 45.6821°N, 66.7894°W, 8.V.2008, R. P. Webster, alder swamp, in moist litter & grass on hummocks near water (9 ♂, 1 ♀, RWC).

###### Collection and habitat data.

The holotype of *Philhygra sinuipennis* from Newfoundland was collected from among litter and stones on a sandy lakeshore (Klimaszewski et al. 2011). The specimens from New Brunswick were sifted from moist litter and grass on hummocks surrounded by water in an alder swamp. The adults were captured during early May in New Brunswick.

###### Distribution in Canada and Alaska.

**NB,** NF (Klimaszewski et al. 2011).

##### Philhygra varula

Casey, 1906**

http://species-id.net/wiki/Philhygra_varula

**Map 34.**
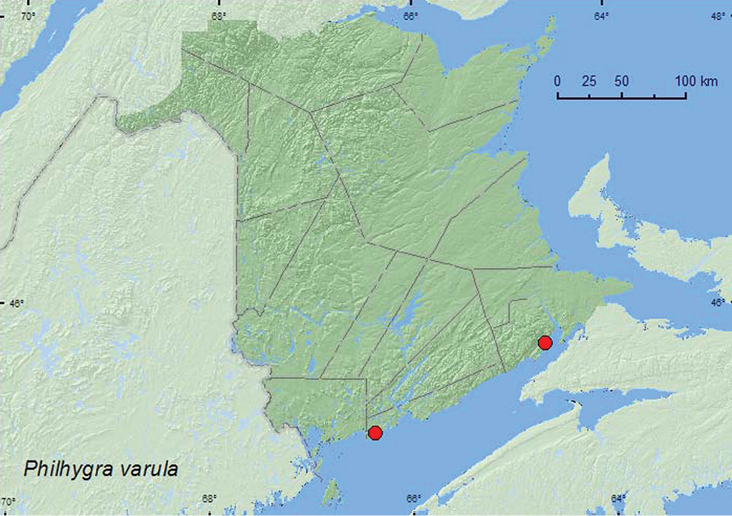
Collection localities in New Brunswick, Canada of *Philhygra varula*.

Map 34 illustrations in Klimaszewski et al. (2011)

###### Material examined.

**New Brunswick, Albert Co.**, Shepody N.W.A., Mary’s Point Section, 45.7321°N, 64.6765°W, 17.V.2004, R. P. Webster, freshwater marsh adjacent to salt marsh, under litter on drift wood (large log) (2 ♀, RWC). **Saint John Co.** Dipper Harbour, 45.1169°N, 66.3771°W, 15.V.2006, R. P. Webster, upper margin sea beach, in decaying sea wrack under alders (2 ♀, RWC); same locality and collector but 45.1182°N, 66.3790°W, 28.V.2010, R. P. Webster, upper margin of salt marsh, in grass litter in seepage area with *Carex* and *Spartina patens* (3 ♂, 3 ♀, RWC).

###### Collection and habitat data.

This species has been reported from under lakeshore debris and in rotting mushrooms in Newfoundland (Klimaszewski et al. (2011) and captured in an estuary above the tidal zone, under stones, and along a stream in silt, gravel, and leaf litter at other localities (Lohse et al. 1990). In New Brunswick, this species was associated with coastal habitats. Adults were collected from under litter resting on a large log (drift wood) in a freshwater marsh adjacent to a salt marsh, along the upper margin of a salt marsh in grass litter in a seepage area with *Carex* and *Spartina patens*, and in decaying sea wrack under alders on the upper margin of a sea beach. Adults were collected during May in New Brunswick.

###### Distribution in Canada and Alaska.

QC, **NB**, LB, NF (Lohse et al. 1990; Klimaszewski et al. 2011).

##### Boreophila eremita

(Rey, 1866)**

http://species-id.net/wiki/Boreophila_eremita

**Map 35.**
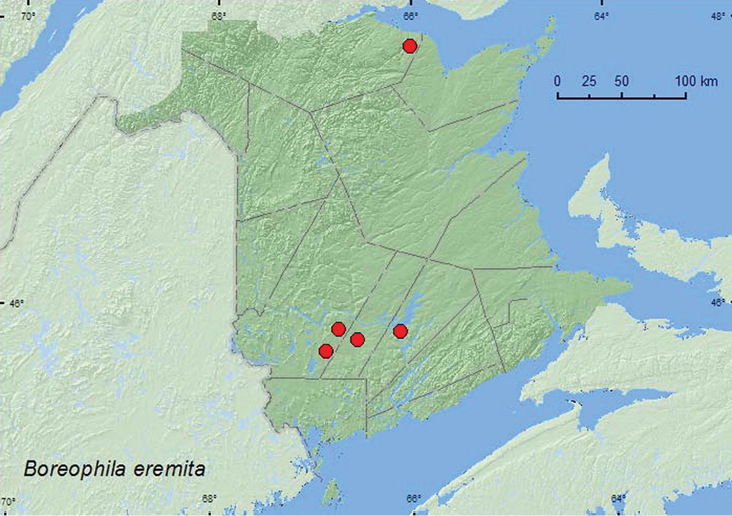
Collection localities in New Brunswick, Canada of *Boreophila eremita*.

Map 35 illustrations in Klimaszewski et al. (2011)

###### Material examined.

**New Brunswick, Queens Co.**, W of Jemseg near “Trout Creek”, 45.8255°N, 66.1174°W, 1.VII.2008, R. P. Webster, seasonally flooded marsh, treading vegetation near margin of pool (1 ♀, RWC). **Restigouche Co.**, Jacquet River Gorge P.N.A., 47.8221°N, 66.0082°W, 13.V.2010, R. P. Webster, margin of *Carex* marsh, in leaf litter and grass litter under shrubs (1 ♀, NBM). **Sunbury Co.**, near Sunpoke Lake, 45.7658°N, 66.5546°W, 3.VII.2008, R. P. Webster, red oak forest near flooded marsh, in leaf litter (1 ♂, RWC). **York Co.** Rt. 645 at Beaver Brook, 45.6860°N, 66.8668°W, 6.V.2008, R. P. Webster, *Carex* marsh, in (woody) litter at base of dead red maple (2 ♂, 3 ♀, RWC); Charters Settlement, 45.8395°N, 66.7391°W, 6.V.2008, R. P. Webster, mixed forest, in flight on warm 20°C afternoon (1 ♀, RWC).

###### Collection and habitat data.

*Boreophila eremita* was collected from various marsh habitats in New Brunswick. Adults were collected by treading vegetation in a seasonally flooded marsh, sifted from leaf litter and grass litter under alders in a *Carex* marsh, sifted from woody litter at the base of a dead red maple in a *Carex* marsh (probably an overwintering site), and sifted from leaf litter in a red oak marsh surrounded by a completely flooded marsh. One individual was collected with an aerial net during a warm (20°C) afternoon. Adults were captured during May and July in New Brunswick. No other habitat data are available from Canada and Alaska (Klimaszewski et al. 2011).

###### Distribution in Canada and Alaska.

AK, YT, **NB,** NF (Gouix and Klimaszewski 2007; Klimaszewski et al. 2011). This species was reported by Klimaszewski et al. (2011) as occurring in New Brunswick, however, there are no previously published records of its occurrence in the province.

##### Thamiaraea brittoni

(Casey, 1911)**

http://species-id.net/wiki/Thamiaraea_brittoni

**Map 36.**
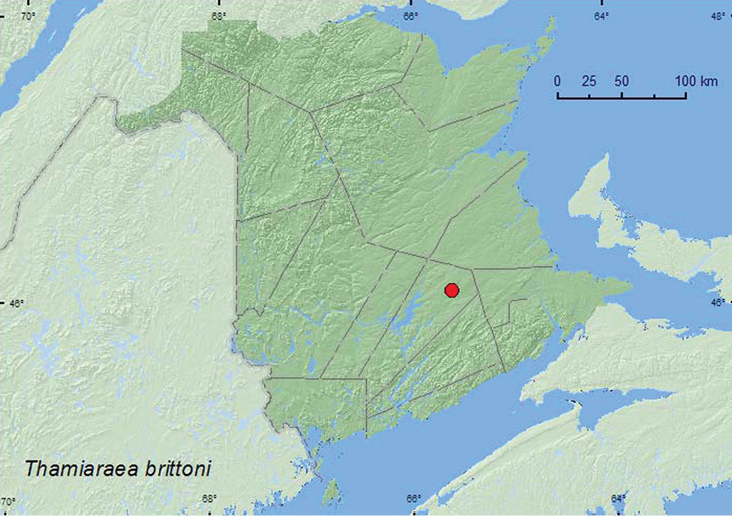
Collection localities in New Brunswick, Canada of *Thamiaraea brittoni*.

Map 36 illustrations Hoebeke (1988) (under synonymic name of *Thamiaraea lira* Hoebeke) Gusarov (2003)

###### Material examined.

**New Brunswick, Queens Co.,** Cranberry Lake P.N.A., 46.1125°N, 65.6075°W, 25.VI-1.VII.2009, R. Webster and M.-A. Giguère coll., red oak forest, Lindgren funnel trap (1 ♂, RWC); same locality data and forest type, 12-26.VII.2010, R. Webster & C. MacKay, Lindgren funnel trap (1 ♂, RWC).

###### Collection and habitat data. 

The two males were captured in a red oak forest during June and July using Lindgren funnel traps.

###### Distribution in Canada and Alaska. 

ON, QC, **NB** (Gusarov 2003; Gouix and Klimaszewski 2007). Gusarov (2003) reported this species as widely distributed in eastern USA.

#### Tribe Falagriini Mulsant & Rey, 1873

##### Cordalia obscura

Gravenhorst 1802**

http://species-id.net/wiki/Cordalia_obscura

**Map 37.**
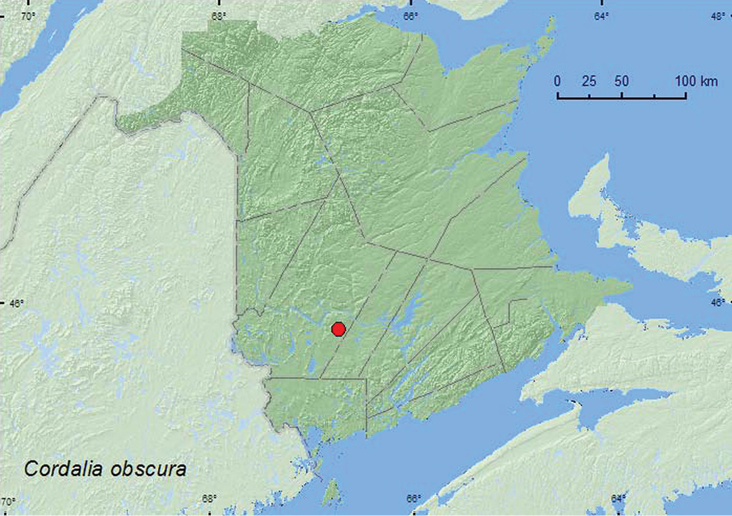
Collection localities in New Brunswick, Canada of *Cordalia obscura*.

Map 37 illustration in Hoebeke (1985) Gouix and Klimaszewski (2007)

###### Material examined. 

**New Brunswick, York Co.**, Charters Settlement, 45.8395°N, 66.7391°W, 20.VI.2008, 9.IX.2009, 17.V.2010, 18.IX.2010, R. P. Webster, mixed forest, in decaying (moldy) corncobs and cornhusks (1 ♀, 3 sex undetermined, RWC).

###### Collection and habitat data.

Hoebeke (1985) reported this adventive species in North America from various kinds of organic debris including grass clippings (sifting), from a Berlese sample of decaying vegetation and compost, from rotten bracket fungus, garden soil, and a trap baited with bacon. The specimens from New Brunswick were collected from decaying, moldy corncobs and cornhusks during May, June, and September.

###### Distribution in Canada and Alaska.

ON, QC, **NB** (Hoebeke 1985).

##### Falagria sulcata

(Paykull, 1789)**

http://species-id.net/wiki/Falagria_sulcata

**Map 38.**
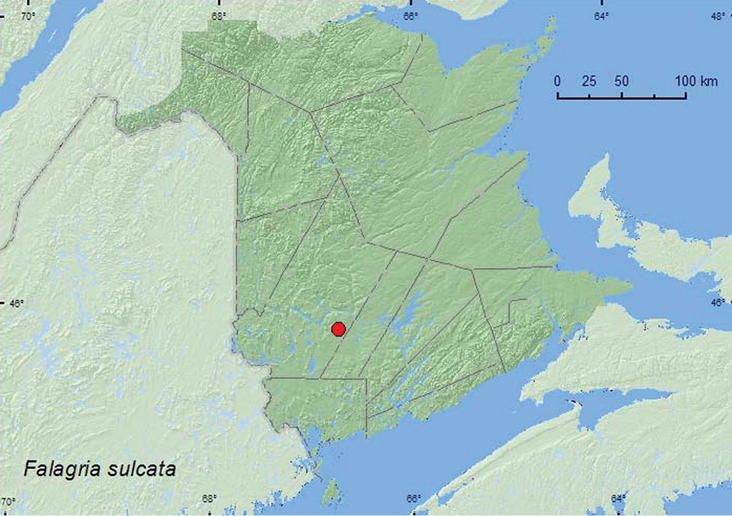
Collection localities in New Brunswick, Canada of *Falagria sulcata*.

Map 38 illustrations in Hoebeke (1985)

###### Material examined.

**New Brunswick, York Co.**, Charters Settlement, 45.8395°N, 66.7391°W, 16.X.2004, R. P. Webster, mixed forest, in compost (decaying vegetables) (2 sex undetermined, RWC).

###### Collection and habitat data.

In North America, this adventive species was reported from a haystack byHoebeke (1985). In New Brunswick, adults were collected from decaying, moldy corncobs and cornhusks. The two adults were captured during October.

###### Distribution in Canada and Alaska.

AB, ON, QC, **NB** (Hoebeke 1985).

## Supplementary Material

XML Treatment for Aleochara rubripennis

XML Treatment for Gnathusa minutissima

XML Treatment for Oxypoda orbicollis

XML Treatment for Phloeopora oregona

XML Treatment for Brachyusa helenae

XML Treatment for Gnypeta atrolucens

XML Treatment for Tachyusa americanoides

XML Treatment for Tachyusa obsoleta

XML Treatment for Gyrophaena nana

XML Treatment for Gyrophaena neonana

XML Treatment for Gyrophaena caseyi

XML Treatment for Gyrophaena nanoides

XML Treatment for Gyrophaena michigana

XML Treatment for Gyrophaena uteana

XML Treatment for Gyrophaena wisconsinica

XML Treatment for Leptusa gatineauensis

XML Treatment for Leptusa (Boreoleptusa) canonica

XML Treatment for Silusa langori

XML Treatment for Acrotona smithi

XML Treatment for Acrotona sequestralis

XML Treatment for Atheta (Atheta) circulicollis

XML Treatment for Atheta (Dimetrota) pseudomodesta

XML Treatment for Atheta (Dimetrota) terranovae

XML Treatment for Atheta (Microdota) pseudosubtilis

XML Treatment for Clusiota impressicollis

XML Treatment for Hydrosmecta pseudodiosica

XML Treatment for Hydrosmecta newfoundlandica

XML Treatment for Tomoglossa decora

XML Treatment for Liogluta aloconotoides

XML Treatment for Lypoglossa angularis obtusa

XML Treatment for Philhygra jarmilae

XML Treatment for Philhygra luridipennis

XML Treatment for Philhygra sinuipennis

XML Treatment for Philhygra varula

XML Treatment for Boreophila eremita

XML Treatment for Thamiaraea brittoni

XML Treatment for Cordalia obscura

XML Treatment for Falagria sulcata
